# Comparative Genomic and Proteomic Analyses of Three Widespread *Phytophthora* Species: *Phytophthora chlamydospora*, *Phytophthora gonapodyides* and *Phytophthora pseudosyringae*

**DOI:** 10.3390/microorganisms8050653

**Published:** 2020-04-30

**Authors:** Jamie McGowan, Richard O’Hanlon, Rebecca A. Owens, David A. Fitzpatrick

**Affiliations:** 1 Department of Biology, Maynooth University, Maynooth, W23 F2H6 Co. Kildare, Ireland; rebecca.owens@mu.ie (R.A.O.); david.fitzpatrick@mu.ie (D.A.F.); 2 Lonsdale Institute for Human Health Research, Maynooth University, Maynooth, W23 F2H6 Co. Kildare, Ireland; 3 Agri-Food & Biosciences Institute (AFBI), Belfast BT9 5PX, UK; richard.ohanlon@afbini.gov.uk

**Keywords:** comparative genomics, effectors, extracellular proteome, secretome, genome evolution, mass spectrometry, phylogenomics, plant pathogen, phytophthora proteomics

## Abstract

The *Phytophthora* genus includes some of the most devastating plant pathogens. Here we report draft genome sequences for three ubiquitous *Phytophthora* species—*Phytophthora chlamydospora*, *Phytophthora gonapodyides*, and *Phytophthora pseudosyringae*. *Phytophthora pseudosyringae* is an important forest pathogen that is abundant in Europe and North America. *Phytophthora chlamydospora* and *Ph. gonapodyides* are globally widespread species often associated with aquatic habitats. They are both regarded as opportunistic plant pathogens. The three sequenced genomes range in size from 45 Mb to 61 Mb. Similar to other oomycete species, tandem gene duplication appears to have played an important role in the expansion of effector arsenals. Comparative analysis of carbohydrate-active enzymes (CAZymes) across 44 oomycete genomes indicates that oomycete lifestyles may be linked to CAZyme repertoires. The mitochondrial genome sequence of each species was also determined, and their gene content and genome structure were compared. Using mass spectrometry, we characterised the extracellular proteome of each species and identified large numbers of proteins putatively involved in pathogenicity and osmotrophy. The mycelial proteome of each species was also characterised using mass spectrometry. In total, the expression of approximately 3000 genes per species was validated at the protein level. These genome resources will be valuable for future studies to understand the behaviour of these three widespread *Phytophthora* species.

## 1. Introduction

*Phytophthora* are filamentous, osmotrophic eukaryotes that morphologically resemble fungi but belong to the Oomycota class within the Stramenopila [1]. *Phytophthora* species include some of the most destructive plant pathogens, including devastating pathogens of important crops, ornamental plants, and forests. The number of identified *Phytophthora* species is rapidly increasing and currently includes more than 180 provisionally named species [2]. In recent years, the genomes of several *Phytophthora* species have been sequenced which has increased our understanding of *Phytophthora* evolution and pathology [3,4,5,6,7,8,9].

As osmotrophs, *Phytophthora* species secrete numerous classes of hydrolytic enzymes including carbohydrate-active enzymes (CAZymes) and proteases that digest complex extracellular substrates into simpler subunits for nutritional sources [10]. *Phytophthora* genomes also encode large arsenals of effector proteins that facilitate infection [11,12]. Effectors are divided into two broad classes based on where they localise. Apoplastic effectors are secreted by the pathogen and act in the host’s extracellular environment [13]. Apoplastic effector proteins encompass a broad range of functions, including hydrolytic enzymes that degrade plant cell walls, facilitating hyphal penetration. Other apoplastic effector families include elicitins, necrosis-inducing proteins (NLPs), and Pcf phytotoxins that play roles in inducing host cell death [14]. *Phytophthora* also secrete cytoplasmic effectors that are translocated into host cells, namely the RxLR and Crinkler (CRN) families of effectors [13].

To date, most large-scale analyses of oomycete secretomes or effector arsenals have used in silico bioinformatics-based approaches applied to genome sequences to predict protein sequences bearing signal peptides. However, mass spectrometry is a powerful technique that can be used to characterise the extracellular proteomes of filamentous plant pathogens. It can also be used to validate the secretion of proteins that have been computationally predicted to be secreted as well as identifying extracellular proteins that lack conventional signal peptides, which would otherwise be overlooked. To date, proteomic studies of oomycete extracellular proteins are limited. Liquid chromatography tandem mass spectrometry (LC-MS/MS) analysis of the potato late blight pathogen *Ph. infestans* led to the identification of 283 extracellular proteins, many of which are members of known effector families such as RxLRs, CRNs, elicitins and NLPs [15]. Similarly, LC-MS/MS analysis of *Ph. plurivora*, an important forest pathogen, led to the detection of 272 secreted proteins, including important effectors and cell-wall degrading enzymes [16]. Proteomic studies of oomycete hyphae have also revealed important insights into metabolism, pathogenicity, and species-specific proteins [17,18,19].

Here, we report draft genome sequences for three globally widespread *Phytophthora* species—*Phytophthora chlamydospora*, *Phytophthora gonapodyides* and *Phytophthora pseudosyringae*. We selected these species because they are (i) widespread *Phytophthora* species in western Europe, (ii) have broadly different lifestyles, and (iii) are not regulated organisms under plant health legislation in Europe. Furthermore, all three species belong to clades that are generally underrepresented in terms of the amount of genomic data available for those clades. *Phytophthora pseudosyringae* belongs to clade 3 and is an important forest pathogen with a broad host range that is widespread across Europe and America [20,21,22]. *Phytophthora pseudosyringae* has been reported to cause collar, root or stem rot in a wide range of host species including European beech (*Fagus sylvatica*), southern beech (*Nothofagus* spp.), oak (*Quercus* spp.), alder (*Alnus* spp.), Japanese larch (*Larix kaempferi*), sweet chestnut (*Castanea sativa*), horse chestnut (*Aesculus hippocastanum*), and shrubs (*Vaccinium myrtillus*) [23,24,25,26,27]. In a recent metabarcoding study, *Ph. pseudosyringae* was the most abundant *Phytophthora* species detected in Britain [28].

*Phytophthora chlamydospora* and *Ph. gonapodyides* both belong to clade 6, which contains species that are primarily associated with streams and riparian ecosystems, and have evolved to tolerate higher temperatures, facilitating survival throughout seasonal changes [29,30]. *Phytophthora gonapodyides* is reported to be the most widely distributed *Phytophthora* species in natural habitats worldwide, followed by *Ph. chlamydospora* [23,31]. *Phytophthora gonapodyides* was the third most abundant *Phytophthora* species detected via metabarcoding in Britain [28]. *Phytophthora chlamydospora* was also frequently detected. *Phytophthora chlamydospora*, previously informally known as *Phytophthora* taxon Pgchlamydo, was formally designated as a species in 2015 [31]. Similar to other clade 6 *Phytophthora* species, both *Ph. chlamydospora* and *Ph. gonapodyides* are thought to exhibit primarily saprophytic lifestyles, maintaining their populations in natural ecosystems by colonising plant litter or through pathogenesis of fine-roots [30]. However, both species are known to act as opportunistic pathogens, with reports of *Ph. chlamydospora* causing disease on *Rhododendron*, *Viburnum tinus,* sour cherry (*Prunus cerasus*), almond (*Prunus dulcis*), walnut (*Juglans regia*), and evergreen nursery stock [22,32,33,34,35,36]. Similarly, *Ph. gonapodyides* has been detected causing disease on European beech (*F*. *sylvatica*), *Rhododendron*, oak (*Quercus* spp.), apple (*Malus* spp.), walnut (*J. regia*) and tanoak (*Notholithocarpus* spp.) [23,37,38,39,40].

Using a combination of bioinformatics, comparative genomics and mass spectrometry-based approaches, we provide a comprehensive characterisation of the nuclear genomes, mitochondrial genomes, putative effector arsenals, extracellular proteomes, and mycelial proteomes of *Ph. chlamydospora*, *Ph. gonapodyides,* and *Ph. pseudosyringae.* The genomic and proteomic data reported herein represent valuable new resources to study the pathogenicity mechanisms of these widespread phytopathogens and further elucidate *Phytophthora* genome evolution.

## 2. Materials and Methods

### 2.1. Isolation and Culturing

The details of the isolates used in this study are provided in Table 1. Methods for the isolation of the isolates and tentative morphological identification have been reported previously [41,42]. Briefly, this involved plating the samples onto P_5_ARP[H] agar (Cornmeal agar with antibiotics [43]) and incubating at room temperature for two weeks. These agar plates were checked at least every three days for *Phytophthora* like structures (e.g., sporangia, *coenocytic* hyphae, oospores). Mycelia from the edge of *Phytophthora* like cultures was then transferred to Carrot Piece Agar (CPA) [44] plates and incubated at room temperature, studied morphologically and compared to *Phytophthora* taxonomy reference text [45,46] (www.phytophthoradb.org). Identification of the isolates based on BLAST [47] searches of the ITS region was carried out by O’Hanlon et al. (2016). Isolates were stored on CPA agar slopes under sterile mineral oil at room temperature between 2016 and 2018. From 2018 onwards, isolates were routinely cultured on 10% clarified V8 juice (cV8) agar in the dark at 20 °C for *Ph. pseudosyringae* and 25 °C for *Ph. chlamydospora* and *Ph. gonapodyides*. Species identities were confirmed based on a combination of β-tub, COX1, COX2, ITS, and Rps10 markers retrieved from the whole genome sequences and compared to reference sequences from PhytophthoraDB (http://www.phytophthoradb.org) and NCBI GenBank using BLASTn [47].

### 2.2. DNA Extraction and Sequencing

To prepare mycelium for DNA extraction, *Phytophthora* cultures were grown in 10% cV8 liquid medium for 5 days. Mycelia were harvested using Miracloth, washed with sterile distilled water, flash-frozen in liquid nitrogen, lyophilized and stored at −80 °C until used for DNA extraction. Lyophilized mycelium was ground to a fine powder with a mortar and pestle under liquid nitrogen. DNA was extracted by transferring 20–40 mg of ground mycelia to a tube containing 800 µL of extraction buffer (0.2 M Tris-HCl, 0.25 M NaCl, 25 mM EDTA and 0.5% SDS) and 2 µL Proteinase K (20 mg/mL; Qiagen, Redwood City, CA, USA). Samples were incubated at 55 °C for 30 min. Samples were then treated with 3 µL RNase A (10 mg/mL; Thermo Fisher Scientific, Waltham, MA, USA) and incubated at 37 °C for 30 min. 800 µL of 24:1 chloroform:isoamyl alcohol was added to samples, mixed by inversion and centrifuged for 10 min at 13,000× *g*. The upper phase was transferred to a new tube and the chloroform step was repeated. DNA was precipitated by the addition of ½ volume of 5 M ammonium acetate and 2 volumes of 100% ethanol, followed by overnight incubation at −20 °C. Precipitated DNA was pelleted by centrifugation for 15 min at 13,000× *g*. The DNA pellet was washed twice, first with 70% ethanol and then with 100% ethanol. DNA was air-dried and resuspended in 70 µL TE buffer (10 mM Tris-HCl, 0.5 mM EDTA). DNA purity was assessed using a Nanodrop spectrophotometer (Thermo Fisher Scientific) based on the 260/280 and 260/230 absorbance ratios. DNA concentration was determined using a Qubit fluorometer with the dsDNA BR kit (Invitrogen, Carlsbad, CA, USA). DNA quality was assessed via agarose gel electrophoresis on a 1% agarose gel. DNA library construction and paired-end sequencing were carried out by BGI Tech Solutions Co., Ltd. (Hong Kong, China) on the Illumina HiSeq X Ten platform. Sequenced reads were deposited on the NCBI Sequence Read Archive (accessions: SRR10849951 for *Ph. chlamydospora*, SRR10849950 for *Ph. gonapodyides* and SRR10849937 for *Ph. pseudosyringae*).

### 2.3. Genome Assembly

Genome sizes and heterozygosity levels were estimated by generating *k*-mer count histograms of sequence reads with Jellyfish [48], which were used as input for GenomeScope [49]. De novo genome assembly was performed using SPAdes (v3.13.1) [50]. Assemblies were further scaffolded using SSPACE (v3.0) [51] and gaps were closed with GapFiller (v1.10) [52]. Scaffolds with low coverage (less than 30×) were manually assessed for contamination by BLAST searches against the NCBI database and removed if they had a top hit outside the Oomycota. Assembly metrics were calculated using Quast (v5.0.2) [53]. BUSCO (v3) [54] was used to assess the gene space completeness of assemblies with the alveolata–stramenopiles dataset of BUSCOs. De novo repeat family identification and repeat masking were performed using RepeatModeler (v2.0) [55] and RepeatMasker (v4.1.0). Mitochondrial genomes were de novo assembled separately using NOVOPlasty (v3.7) [56] and visualised using OGDRAW (v1.3.1) [57].

### 2.4. Gene Annotation

Gene models were predicted using BRAKER2 [58] with the ProHint pipeline [59]. In brief, initial gene sets were predicted using GeneMark-ES (v4.46) [60]. Homologs of the initial gene predictions were identified using Diamond [61] searches against a database of 14 Peronosporales proteomes containing 267,298 proteins (Supplementary Table S1). Intron hints were generated by performing spliced alignments using Spaln2 [62] with the ProtHint pipeline [59]. GeneMark-EP [59] was trained with the intron hints and used to generate another gene set. The GeneMark-EP predictions, along with the intron hints, were then used to train Augustus [63] to generate the final gene sets. Completeness of gene sets were assessed using BUSCO v3.

Genes were functionally annotated using InterProScan (v5.39-77.0) [64] and eggNOG-mapper (v2) [65]. Secreted proteins and transmembrane proteins were predicted using SignalP v3 [66] and TMHMM (v2.0) [67], respectively. SignalP v3 was implemented instead of earlier or later versions of the software as previous studies have found v3 to be more sensitive in predicting oomycete signal peptides [68]. For a protein to be considered secreted, it had to have a positive prediction from SignalP, an HMM S probability value ≥0.9, an NN Y_max_ score of ≥0.5, an NN D score of ≥0.5, and no transmembrane domains downstream of the predicted signal peptide cleavage site. These criteria permitted comparisons to previous studies [12,68,69]. Proteins predicted to be secreted were submitted to ApoplastP [70] to predict if they are localised to the plant apoplast. CAZymes were annotated using dbCAN2 [71]. Homologs of experimentally verified effector proteins were identified by performing BLASTp searches [47] against the pathogen-host interaction database (PHI-Base Release 4.8) [72] with an *E* value cut-off of 1e^−20^.

Gene families were identified by performing all-versus-all BLASTp [47] searches with an *E* value cut-off of 1e^−10^, followed by Markov clustering using MCL [73] with an inflation value of 1.5. Tandemly duplicated genes were identified using BLASTp [47]. Tandem clusters were defined as two or more adjacent genes that hit each other in a BLASTp search with an *E* value cut-off of 1e^−10^ and highest-scoring pair (HSP) length greater than half the length of the shortest sequence. Enrichment analyses were preformed using Fisher’s exact test. Gene ontology enrichment analyses were performed using Fisher’s exact test with Benjamini–Hochberg correction for multiple testing using GOATOOLS [74]. Corrected *p*-values < 0.05 were considered significant.

### 2.5. Identification of Cytoplasmic Effectors

RxLRs were classified using four methods as in McGowan and Fitzpatrick (2017) [12]. (i) The Win method—proteins must contain a signal peptide with a predicted cleavage site within the first 30 amino acids and an RxLR motif within residues 30–60 [75]. (ii) HMM method—hidden Markov model (HMM) searches were performed with HMMER (v3.2.1) [76] against all proteins predicted to be secreted using the “cropped.hmm” HMM profile constructed by Whisson et al. (2007) [77]. Hits with a bit score >0 were retained. (iii) Regex method—proteins must contain a signal peptide between residues 10 to 40 and an RxLR motif within the following 100 residues followed by the EER motif within 40 residues downstream of the RxLR motif, allowing for replacements of E to D and R to K [77]. This search was performed using the regular expression “^.{10,40}.{1,96}R.LR.{1,40}[ED][ED][KR]”. (iv) Homology method—proteins with a positive SignalP prediction were searched using BLASTp against a set of 1207 putative *Phytophthora* RxLRs from *Ph. infestans*, *Ph. ramorum* and *Ph. sojae* [3]. An *E* value cut-off of 1e^−20^ was applied. Secreted proteins that met at least one of these four criteria were considered to be putative RxLRs. Additionally, an HMM search was performed on all putative RxLRs to determine if they have WY-domains, using the HMM described by Boutemy et al. (2011) [78].

CRNs were identified using the regular expression “^.{30,70}L[FY]LA[RK]”. Proteins with a positive hit from the regular expression search were aligned and an HMM model was constructed. The CRN HMM was then searched against the predicted proteomes using HMMER [76] and all proteins with a bit score >0 were considered the final set of putative CRNs.

### 2.6. Phylogenomics

A dataset of 33 Peronosporales genomes (Supplementary Table S1) was used for phylogenomic analysis. We also included *Pythium ultimum* as an outgroup. BUSCO analysis revealed 208 BUSCO families that are present and single copy in at least 90% of the species (i.e., at least 31 of the 34 species). Each BUSCO family was individually aligned with MUSCLE (v3.8.31) [79] and trimmed using trimAl (v1.4) [80] with the parameter “-automated1” to remove poorly aligned regions. Trimmed alignments were concatenated together resulting in a final supermatrix alignment of 106,315 amino acids. Maximum-likelihood (ML) phylogenetic reconstruction was performed using IQ-TREE (v1.6.12) [81] with the JTT+F+R5 model, which was the best fit model according to ModelFinder [82], and 100 bootstrap replicates were undertaken to infer branch support values. Bayesian analysis was also performed using PhyloBayes MPI (v1.8) [83] with the CAT model. Two independent chains were run for 10,000 cycles and convergence was assessed using bpcomp and tracecomp. A consensus Bayesian phylogeny was generated with a burn-in of 10%. The phylogeny was visualised and annotated using the Interactive Tree of Life (iTOL) [84].

### 2.7. Phylostratigraphy

Phylostratigraphic maps were determined for each species following previously published methods [85,86]. The database constructed by Drost et al. (2015) was retrieved which contains amino acid sequences from 4557 species, including 1787 eukaryotes (883 animals, 364 plants, 344 fungi and 193 other eukaryotes) and 2770 prokaryotes (2511 bacteria and 259 archaea) [85]. Protein sequences from the three *Phytophthora* species sequenced in this study and any publicly available oomycete proteomes were added to the database. The final database comprised 18,084,866 proteins, including 578,493 proteins from 38 oomycete genomes. Each *Phytophthora* protein was then searched against this database using BLASTp [47]. Proteins were assigned to the oldest phylostratum that contained at least one BLAST hit with an *E* value cut-off < 1e^−5^. Genes that did not have a BLAST hit to any other species were considered species-specific (orphans).

### 2.8. Culturing Conditions and Extraction of Phytophthora Extracellular Proteins

Petri dishes containing 15 mL of liquid medium (either 10% V8 broth or 10% cV8 broth) were inoculated with a 10 mm agar plug of *Phytophthora* mycelium cut from the edge of a growing *Phytophthora* colony. Cultures were incubated in the dark, non-shaking for 10 days at their optimum temperatures (20 °C for *Ph. pseudosyringae* and 25 °C for *Ph. chlamydospora* and *Ph. gonapodyides*). Spent growth medium was harvested using a syringe without disturbing the mycelium. Supernatant from four petri dishes were pooled to make up one replicate. Collected supernatant was passed through a 0.2 μm syringe filter, frozen overnight at −20 °C and lyophilized. Lyophilized supernatant was resuspended in minimal volumes of PBS, desalted and concentrated using Amicon Ultra centrifugal filters (Millipore, Billerica, MA, USA) with a 3 kDa cut-off. Samples were clarified by centrifugation at 12,000× *g* for 5 min and brought to 15% (*v*/*v*) trichloroacetic acid (TCA) using 100% TCA. Precipitated proteins were washed twice with ice-cold acetone. Dried protein pellets were resuspended in 6 M Urea, 2 M Thiourea and 0.1 M Tris-HCl pH 8.0. Protein concentration was determined using a Qubit fluorometer (Invitrogen).

### 2.9. Culturing Conditions and Extraction of Phytophthora Mycelial Proteins

*Phytophthora* mycelium was cultured under three growth conditions. (i) Normal–cultures were grown for ten days at their optimum temperatures. (ii) Heat–cultures were grown for 7 days at their optimum temperatures, followed by incubation at 30 °C for three days. (iii) Oxidative stress–cultures were grown for ten days at their optimum temperatures, then exposed to 1 mM H_2_O_2_ for three hours. All cultures were grown in 50 mL of 10% cV8, non-shaking. Mycelia were harvested using Miracloth, washed with sterile distilled water, flash-frozen in liquid nitrogen and stored at −80 °C until used for protein extraction.

To extract proteins, mycelium was ground to a fine powder with a mortar and pestle under liquid nitrogen. 200–300 mg of ground mycelium was resuspended in 400 µL of lysis buffer followed by sonication (Bandelin Sonopuls HD2200 sonicator, Cycle 6, Berlin, Germany, 3 × 10 s, Power 20%). Protein concentration was determined using a Qubit fluorometer (Invitrogen). Protein lysates (0.25 mg/mL) were incubated at 95 °C for 5 min.

### 2.10. Protein Digestion and LC-MS/MS Identification of Phytophthora Proteins

Three independent biological replicates were analysed for each condition. Proteins were reduced and alkylated prior to overnight trypsin digestion as described previously [87,88]. Digestion was terminated by the addition of 1 µL of 100% trifluoroacetic acid (TFA). Sample clean-up was performed using C18 ZipTips^®^ (Millipore), following the manufacturer’s instructions. Shotgun proteomics was performed using an Ultimate 3000 RSLC from Dionex, coupled to a Thermo Scientific Q-Exactive mass spectrometer. Peptide mixtures were separated on a 50 cm EASY-Spray PepMap C18 column with 75 µm diameter (2 µm particle size) using a 10–40 % B gradient (A: 0.1% (*v*/*v*) formic acid, 3% (*v*/*v*) acetonitrile; B: 0.1% (*v*/*v*) formic acid, 80% (*v*/*v*) acetonitrile). Data were acquired for 105 min, at 70,000 resolution for MS and a Top 15 method for MS2 collection.

Protein identification from the data was performed using the Andromeda search engine [89] in MaxQuant [90] with the predicted proteomes for each species as a search database. To account for possible protein contamination from V8 juice medium, we appended the tomato proteome to the default MaxQuant contaminants database. Search parameters were as described in Delgado et al. (2019) [91]. Identified protein groups were filtered using Perseus [92], to remove protein groups that were identified only by site, or had hits to either the contaminants database or the reverse database. Proteins were considered present in a condition if they were identified by 2 or more peptides and detected in at least 2 out of 3 replicates. Proteins were considered unique to a condition if they were not detected in any replicate of any other condition.

### 2.11. Data Deposition

This project has been deposited at DDBJ/EMBL/GenBank as individual WGS BioProjects, with the following BioProject accession numbers: PRJNA599565 (*Ph. chlamydospora*), PRJNA599567 (*Ph. gonapodyides*) and PRJNA599564 (*Ph. pseudosyringae*).

## 3. Results and Discussion

### 3.1. Genome Sequencing and Assembly

**Figure 1:**
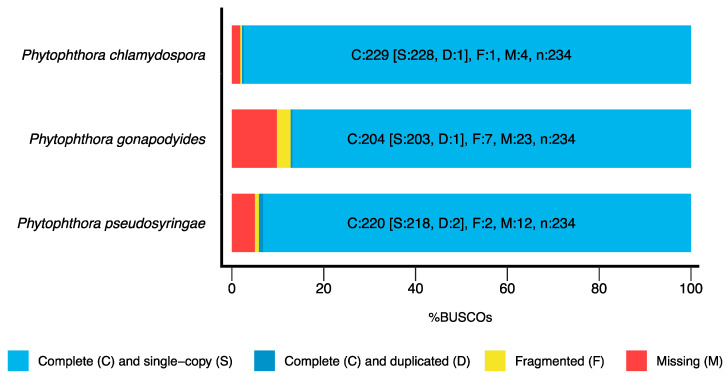
BUSCO analysis of *Ph. chlamydospora*, *Ph. gonapodyides*, and *Ph. pseudosyringae* using the Alveolata-Stramenopiles dataset. BUSCO completeness in all three species is high (97.8%, 87.2% and 94.1%) indicating that the genome assemblies are of high gene space completeness. The lower level of BUSCO completeness in *Ph. gonapodyides* is most likely the result of a fragmented assembly due to high levels of heterozygosity.

Paired-end Illumina sequencing generated approximately 5.2 Gb of sequencing data for each of the three species. Genome sizes and heterozygosity levels were estimated based on K-mer analysis of sequence reads using Jellyfish [48] and GenomeScope [49], which estimated genome sizes of 51.1 Mb, 65.2 Mb and 51.0 Mb (Table 2) for *Ph. chlamydospora*, *Ph. gonapodyides*, and *Ph. pseudosyringae*, respectively. The heterozygosity of *Ph. gonapodyides* was estimated to be 1.88% (Table 2), which was much higher than *Ph. chlamydospora* (0.68%) and *Ph. pseudosyringae* (0.15%) (Table 2), and high compared to other oomycetes which typically have heterozygosity levels less than 1% [93]. De novo genome assembly using SPAdes [50] generated draft genome assemblies with assembly sizes of 45.3 Mb for *Ph. chlamydospora*, 61.1 Mb for *Ph. gonapodyides* and 47.9 Mb for *Ph. pseudosyringae* (Table 2), which compares favourably to the genome sizes estimated by GenomeScope. The *Ph. gonapodyides* assembly was much more fragmented (16,449 scaffolds) than *Ph. chlamydospora* (4077 scaffolds) and *Ph. pseudosyringae* (3627 scaffolds) (Table 2). We expect that this is due to higher levels of heterozygosity found in the *Ph. gonapodyides* genome assembly and due to expansions of repetitive elements (Supplementary Table S2). BUSCO analysis [54] was performed using the Alveolata-Stramenopiles dataset which contains 234 target BUSCO proteins that are expected to be present and single-copy. BUSCO results suggests that the assemblies are of high gene space completeness with BUSCO completeness values of 97.8% for *Ph. chlamydospora*, 87.2% for *Ph. gonapodyides*, and 94.1% for *Ph. pseudosyringae*) (Table 2 and Figure 1). Furthermore, the low number of duplicated BUSCOs suggest that haplotypes were correctly collapsed (Figure 1). De novo repeat annotation using RepeatModeler2 [55] and RepeatMasker led to the identification of 4.1 Mb (9.0%) of repetitive elements in *Ph. chlamydospora*, 9.8 Mb (16.1%) in *Ph. gonapodyides* and 6.4 Mb (13.3%) in *Ph. pseudosyringae* (Table 2). The majority of identified repeats were unclassified or were classified as long terminal repeat (LTR) retroelements (Supplementary Table S2). Overall, the proportions of repetitive elements identified are similar to that of *Ph. parasitica* (8%), *Ph. plurivora* (15%), *Ph. cactorum* (18%) and *Ph. capsici* (21%) [5,6] but less than that of *Ph. sojae* (31%), *Ph. ramorum* (54%) and *Ph. infestans* (74%) [3,94,95].

Gene prediction led to the annotation of 17,872 gene models for *Ph. chlamydospora*, 23,348 for *Ph. gonapodyides* and 17,439 for *Ph. pseudosyringae* (Table 2), with BUSCO completeness scores of 97.5%, 87.2% and 94.4% (Table 2), suggesting high quality gene model annotation. Between 52.5% and 60.2% of proteins were annotated with one or more Pfam domains (Table 2 and Supplementary Table S3). The percentage of the genome assemblies covered by coding sequences were 56.3%, 43.3% and 49.2% for *Ph. chlamydospora*, *Ph. gonapodyides* and *Ph. pseudosyringae*, respectively, which are similar to *Ph. cinnamomi* (43.7%), *Ph. parasitica* (43.0%) and *Ph. sojae* (45.6%) but higher than what is observed in *Ph. ramorum* (33.7%), *Ph. capsici* (38.8%) and *Ph. infestans* (12.36%) [93].

### 3.2. Phylogenomics Analysis

**Figure 2:**
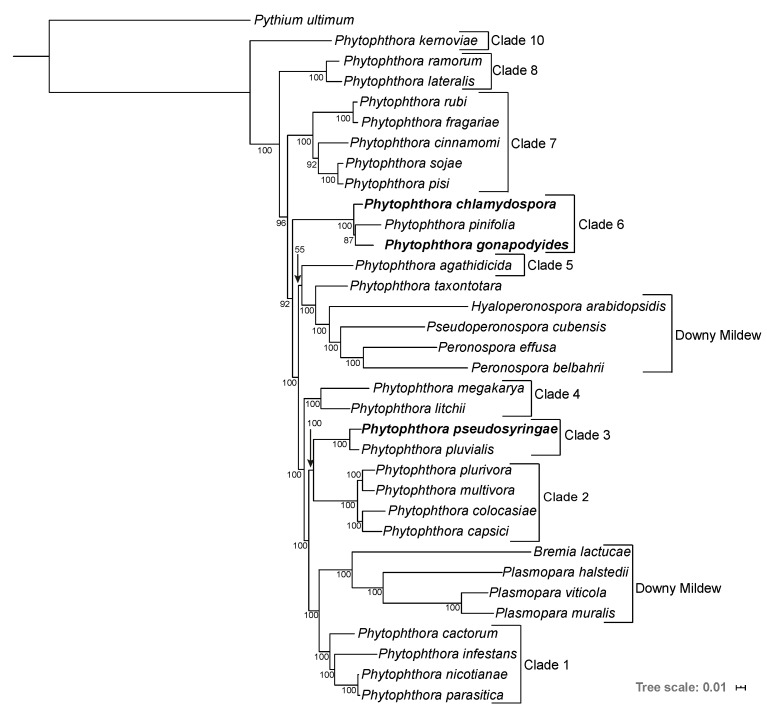
Supermatrix phylogeny of 33 Peronosporales species (208 BUSCO families, 106,315 amino acids). *Py. ultimum* is included as an outgroup. Phylogenomic analyses were performed using both maximum likelihood (IQ-TREE with JTT+F+R5 model) and Bayesian inference (Phylobayes MPI with the CAT model). Both methods inferred phylogenies with identical topologies. Branch lengths are shown. Maximum likelihood bootstrap supports are indicated at all nodes. Bayesian posterior probabilities were 100 for all nodes and are not shown.

A phylogenomic analysis was carried out to determine the phylogenetic relationships of the three *Phytophthora* species using the genome sequences of 33 Peronosporales species and *Py. ultimum* as an outgroup (Supplementary Table S1). A supermatrix alignment was constructed from 208 highly conserved BUSCO families. Maximum Likelihood (ML) and Bayesian phylogenetic reconstruction was undertaken on the supermatrix. Both ML and Bayesian methods resulted in phylogenies with identical topologies and most nodes had maximum Bootstrap Support (BP) or maximum Bayesian Posterior Probabilities (BPP) (Figure 2). All species were placed into their expected clades, and overall the placement of each species is in broad agreement with previous studies [93,96,97]. We also recovered the polyphyly of the downy mildews (Figure 2). Interestingly, the branch lengths of the Downy Mildew species are longer relative to the other Peronosporales species presented indicating higher levels of genetic divergence (Figure 2). *Ph. pseudosyringae* was placed as sister to *Ph. pluvialis*, which is also a clade 3 species, with maximum support from both ML and Bayesian methods (Figure 2). *Phytophthora gonapodyides* was placed as sister to *Ph. pinifolia*, to the exclusion of *Ph. chlamydospora,* with 87% BP from the ML phylogeny and maximum support from the Bayesian phylogeny (Figure 2). We note that this disagrees with previous phylogenies based on ITS sequences [31] and four concatenated mitochondrial loci [98], both of which group *Ph. chlamydospora* and *Ph. pinifolia* as being more closely related. Some markers, including the ITS sequence, are known to be identical or nearly identical between members of *Phytophthora* clade 6b, which *Ph. chlamydospora*, *Ph. gonapodyides*, and *Ph. pinifolia* belong to [99]. However, due to the highly conserved nature of these markers, they may not reflect the true phylogenetic relationships between species. Furthermore, phylogenomic approaches are generally considered to be more informative than single gene phylogenies or phylogenies derived from small numbers of genes, as they utilise substantially greater amounts of phylogenetically informative genomic data [100].

Our phylogeny groups *Ph. cinnamomi* with *Ph. sojae* and *Ph. pisi*, to the exclusion of *Ph. fragariae* and *Ph. rubi*, with 92% BP from the ML phylogeny and maximum support in the Bayesian phylogeny (Figure 2). This is in disagreement with a phylogeny based on seven nuclear genetic markers which groups *Ph. sojae*, *Ph. pisi*, *Ph. fragariae*, and *Ph. rubi* together to the exclusion of *Ph. cinnamomi* [2]. However, our phylogeny is in agreement with two separate studies based on seven nuclear loci which group *Ph. cinnamomi* more closely related to *Ph. sojae* [97,98]. We anticipate that differences in topology are due to the inclusion or exclusion of different species in datasets.

### 3.3. Phytophthora Mitochondrial Genomes

**Figure 3:**
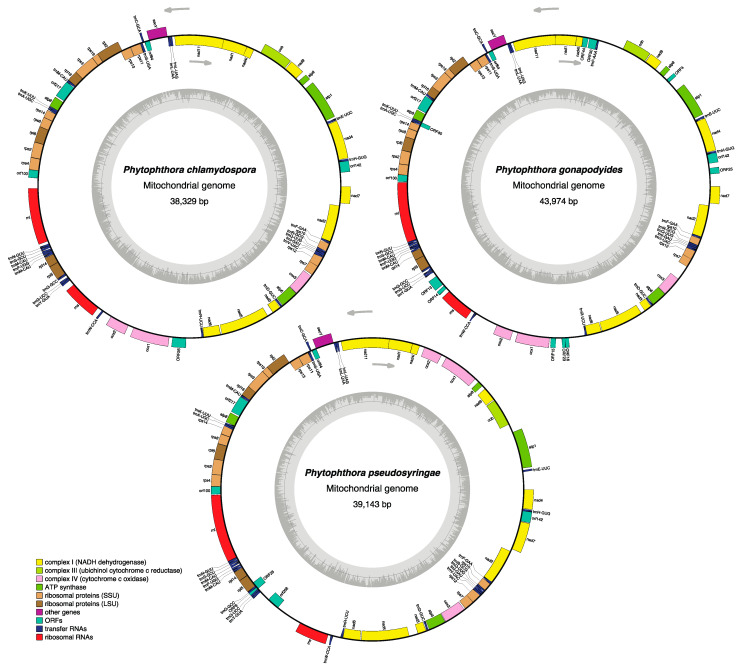
Circular maps of the mitochondrial genomes of *Ph. chlamydospora*, *Ph. gonapodyides* and *Ph. pseudosyringae*. The inner ring shows % GC content. Arrows indicate relative transcriptional orientation. The outer ring shows the predicted genes which are encoded on both strands. All three species are missing the tRNA gene for threonine.

Mitochondrial genomes were assembled and circularised using NOVOPlasty, resulting in mitochondrial genome assemblies sizes of 38.33 Kb for *Ph. chlamydospora*, 43.97 Kb for *Ph. gonapodyides* and 39.14 Kb for *Ph. pseudosyringae* in length (Figure 3), which are similar in size to previously sequenced *Phytophthora* mitochondrial genomes [101]. The overall mitochondrial GC content is also highly similar to other *Phytophthora* species, with 22.5% for *Ph. chlamydospora*, 23.7% for *Ph. gonapodyides* and 22.0% for *Ph. pseudosyringae*. We did not detect any inverted repeats. The gene content of each mitochondrion is similar to that of other *Phytophthora* mitochondrial genomes, including 35 known protein-coding genes (18 respiratory chain proteins, 16 ribosomal proteins, and the import protein secY), two ribosomal RNA genes (rns and rnl) and 25 (*Ph. chlamydospora* and *Ph. pseudosyringae*) or 26 (*Ph. gonapodyides*) transfer RNA genes that specify 19 amino acids (Figure 3 and Supplementary Table S4). As with other oomycetes the tRNA gene for threonine was not located in the mitochondrial genomes of the three species presented here. Unlike animals and fungi, oomycete mitochondria use the standard genetic code [102] The majority of mitochondrial genes had the TAA stop codon except for nad11 which has the TGA stop codon in all three species. Other exceptions include ORF24 (TAG) in *Ph. chlamydospora,* ORFS 13, 23, 40 (TGA) and ORF4 (TAG) in *Ph. gonapodyides* as well as ORFS8&25 in *Ph. pseudosyringae*. Coding regions account for 87.55%, 81.95% and 87.55% of the genomes of *Ph. chlamydospora, Ph. gonapodyides* and *Ph. pseudosyringae* respectively.

Nucleotide alignment of the mitochondrial assemblies revealed that the *Ph. chlamydospora* and *Ph. gonapodyides* mitochondria are collinear (Figure 3 and Supplementary Figure S1). Two inversions are present in the *Ph. pseudosyringae* mitochondrial genome relative to *Ph. chlamydospora* and *Ph. gonapodyides* (Figure 3 and Supplementary Figure S1). We identified a number of open reading frames (ORFs) that are conserved between all three mitochondrial genomes and other *Phytophthora* mitochondrial genomes, including orf64, orf100, orf142 and orf217 (Figure 3 and Supplementary Table S4). The functions of these ORFs are unknown. *Phytophthora pseudosyringae* also shares an additional ORF that is homologous with *Ph. sojae* orf206 [103] (Figure 3). A large number of unique unannotated ORFs were identified in *Ph. gonapodyides* (ORF4, ORF13, ORF14, ORF15, ORF16, ORF23, ORF25, ORF40, ORF44, and ORF50), compared to two in *Ph. pseudosyringae* (ORF8 and ORF25) and only one in *Ph. chlamydospora* (ORF26) (Figure 3).

### 3.4. Bioinformatic Characterisation of Phytophthora Effector Arsenals

Bioinformatic annotation of *Phytophthora* secretomes was performed using SignalP. This analysis predicted 1140, 1291 and 1131 secreted proteins for *Ph. chlamydospora*, *Ph. gonapodyides,* and *Ph. pseudosyringae* respectively (Table 3), accounting for 6.38%, 5.53% and 6.49% of their total genome complement, similar to the number of secreted proteins reported for other *Phytophthora* genomes [12]. ApoplastP predicted that 47.1% to 48.8% of putative secreted proteins localise to the plant apoplast (Table 3). Approximately 20% of all putatively secreted proteins are homologous to experimentally verified effectors in PHI-Base (Table 3). InterProScan [64] was used to annotate putative effector proteins based on conserved Pfam domains known to be implicated in plant pathogenicity. Some of these effectors are discussed below.

Elicitins are secreted proteins that bind sterols and lipids, allowing *Phytophthora* spp. to overcome their inability to synthesise sterols by sequestering sterols from their hosts or environments [104]. Elicitins also act as microbe-associated molecular patterns (MAMPs), triggering host cell death upon recognition by the host plant. Elicitin proteins are usually members of large multi-copy gene families in oomycete genomes [105]. Here, we have identified between 45 and 59 proteins with an elicitin domain (PF00964) for each *Phytophthora* species, of which approximately 78% are predicted to be secreted (Table 3). In contrast to elicitins, necrosis-inducing proteins (NLPs) have a broad taxonomic distribution having been identified in bacteria, fungi and oomycetes [106]. NLPs are known to induce ethylene accumulation and trigger necrosis in dicots [106]. Here, we identified 25, 33 and 22 proteins containing the NLP domain (PF05630) in *Ph. chlamydospora*, *Ph. gonapodyides* and *Ph. pseudosyringae*, respectively, of which 19 (76%), 22 (67%) and 19 (86%) were predicted to be secreted (Table 3). Multiple sequence alignment (not shown) of identified NLPs confirm they are all type 1 NLPs, characterised by the presence of two conserved cysteine residues. Other effectors of interest include the PcF phytotoxins, these are small cysteine-rich proteins that induce plant cell necrosis [107]. PcF phytotoxins appear to be unique to Peronosporales species based on available genomic data. We identified only one protein with the PcF phytotoxin domain (PF09461) in each of the three genomes, however, only the *Ph. gonapodyides* and *Ph. pseudosyringae* copies were predicted to be secreted (Table 3). Transglutaminases are proteins that strengthen structures such as cell walls by facilitating cross-linking between glutamine and lysine residues, conferring resistance to proteolysis [108]. Transglutaminases, such as *Ph. sojae* GP42, can elicit a host immune response upon recognition [109]. We identified 14, 16, and 17 proteins containing the transglutaminase elicitor domain (PF16683) in *Ph. chlamydospora*, *Ph. gonapodyides* and *Ph. pseudosyringae*, respectively (Table 3). Each of the three genomes encode 11 transglutaminase elicitors that are predicted to be secreted (Table 3), which is similar to the number predicted to be secreted by *Ph. cactorum* (15) [6].

The PAN/Apple domain (PF00024, PF14295) is enriched in the secretomes of most oomycete species [12]. This domain is associated with carbohydrate-binding modules, for example, cellulose-binding elicitor lectins (CBEL). Knockdown of a *Ph. parasitica* CBEL with two PAN/Apple domains affected its ability to adhere to cellulosic substrates, such as plant cell walls [110]. We identified 25 proteins with PAN/Apple domains in *Ph. chlamydospora*, 32 in *Ph. gonapodyides* and 20 in *Ph. pseudosyringae* (Table 3), of which 21 (84%), 20 (62.5%) and 15 (75%) were predicted to be secreted (Table 3). 47% of all identified PAN/Apple domain-containing proteins had two or more PAN/Apple domains. Proteins belonging to the cysteine-rich secretory proteins, antigen 5, and pathogenesis-related 1 proteins (CAP) family (PF00188) are also enriched in most oomycete secretomes [12]. *Saccharomyces cerevisiae* CAP family proteins function in sterol binding and export, and are linked to fungal virulence [111]. However, little is known about their involvement in oomycete infection. We identified 34 CAP proteins in *Ph. chlamydospora*, and *Ph. gonapodyides* individually and 31 in *Ph. pseudosyringae* (Table 3), of which 22 (64.7%), 25 (73.5%) and 22 (71.0%) were predicted to be secreted. 

In total, we annotated 18, 13 and 19 proteases bearing signal peptides (aspartyl proteases, papain family cysteine proteases or serine proteases) in *Ph. chlamydospora*, *Ph. gonapodyides* and *Ph. pseudosyringae*, respectively (Table 3). While these proteins are annotated as proteases, they may not be proteolytically active. For example, two *Ph. sojae* proteins GIP1 and GIP2 share significant similarity with trypsin but are proteolytically non-functional and instead inhibit an endo-β-1,3 glucanase from soybean [11]. In a classic example of a co-evolutionary arms race, plant hosts secrete apoplastic proteases to degrade pathogen effectors. To counteract these plant defences, pathogens secrete protease inhibitors to inhibit the host proteases. A total of 13, 15, and 16 proteins were annotated with Kazal-type serine protease inhibitors domains (PF00050) in *Ph. chlamydospora, Ph. gonapodyides,* and *Ph. pseudosyringae*, of which 12 (92.3%), 11 (73.3%) and 10 (62.5%) were predicted to be secreted (Table 3). We also identified 4 Cathepsin propeptide inhibitors (PF08246) in each genome (Table 3), 2 were predicted to be secreted in *Ph. chlamydospora*, 1 in *Ph. gonapodyides* and 3 in *Ph. pseudosyringae* (Table 3).

We also annotated a large number of proteins putatively involved in the breakdown or binding of exogenous carbohydrates, such as plant cell walls, including cellulases, cellulose-binding proteins, cutinases, lytic polysaccharide mono-oxygenases, and pectin modifying enzymes (Table 3). Pectin modifying enzymes were the most numerous and include pectate lyases, pectinesterases and pectin acetylesterases. Pectate lyases cleave pectin, a major component of plant cell walls. Pectinesterases catalyse the de-esterification of pectin, while pectin acetylesterases deacetylate pectin, making the pectin backbone more accessible to pectate lyases [112,113]. In total, we identified 47 pectin modifying enzymes in *Ph. chlamydospora*, 38 in *Ph. gonapodyides*, and 52 in *Ph. pseudosyringae* (Table 3), of which 33 (70.2%), 22 (57.9%) and 37 (71.2%) are predicted to be secreted, suggesting a putative role in the breakdown of plant cells.

RxLR effectors are named due to the highly conserved RxLR motif found in their N-terminus which act as a trafficking motif, signalling the effectors to be delivered into plant cells [77]. The RxLR motif is followed by an EER motif in many RxLR effectors [77]. RxLR C-terminal domains are typically highly divergent although many contain one or more “WY” domains [114]. Many RxLRs are expressed early in infection and play roles in the suppression of host immune responses [115]. However, the function of most RxLRs is unknown and many have been shown to localise to diverse subcellular locations within plant host cells [116]. RxLRs were identified using a combination of four independent criteria (see methods). For *Ph. chlamydospora*, 93 proteins had a hit according to the Win method, 68 with the Regex method, 64 with the HMM method and 81 with the homology method (Supplementary Table S5). In total, across the four methods, 132 unique putative RxLRs were identified for *Ph. chlamydospora* (Table 3), of which 34 had hits to the WY-fold HMM (Supplementary Table S5). For *Ph. gonapodyides*, 96 putative RxLRs were identified according to the Win method, 63 with the Regex method, 68 with the HMM method and 74 with the homology method (Supplementary Table S5). In total, 132 unique putative RxLRs were identified using the four methods (Table 3), 39 of which had hits to the WY-fold HMM (Supplementary Table S5). For *Ph. pseudosyringae*, 125 proteins were designated as putative RxLR effectors based on the Win method, 99 with the Regex method, 101 with the HMM method and 124 with the homology method (Supplementary Table S5). In total, across the four methods, 186 unique proteins were annotated as putative RxLRs (Table 3), of which 61 had hits from the WY-fold HMM (Supplementary Table S5). The number of putative RxLRs identified in *Ph. pseudosyringae* is similar to its clade 3 relative *Ph. pluvialis* (181) [8,12].

CRNs are modular proteins that contain highly conserved N-terminal domains containing a signal peptide and an “LxLFLAK” motif that mediates translocation into host cells [117]. CRNs are named after their crinkling and necrosis-inducing activity in leaves [118]. CRNs were identified using a combination of regular expression searches and HMM searches. The number of CRNs identified for each species is similar. In total, 77 putative CRNs were identified in *Ph. chlamydospora*, 80 in *Ph. gonapodyides* and 90 in *Ph. pseudosyringae* (Table 3). Similar to what has been observed for other oomycetes [5,6,12], only a small proportion of identified CRNs have a positive SignalP prediction, with 28 (36.4%) in *Ph. chlamydospora*, 18 (22.5%) in *Ph. gonapodyides* and 37 (41.1%) in *Ph. pseudosyringae* (Table 3).

### 3.5. Carbohydrate Active Enzymes

Supplemental to the InterProScan analysis above, a more detailed analysis of CAZymes was performed using dbCAN2 [71]. This led to the identification of 483 putative CAZymes in *Ph. chlamydospora*, 487 in *Ph. gonapodyides* and 453 in *Ph. pseudosyringae* (Table 4 and Supplementary Table S6), of which, 213 (44.1%), 179 (36.8%) and 194 (42.8%) are predicted to be secreted (Table 4 and Supplementary Table S6). Of the identified CAZymes, glycoside hydrolases are the most numerous. Each species encodes between 210 and 243 glycoside hydrolases, of which 51 to 62% are predicted to be secreted (Table 4). Identified glycoside hydrolases belonged to 44 families/subfamilies (Supplementary Table S6). In total 33 to 38 polysaccharide lyases were identified for each species (Table 4), belonging to three families, PL1_4 (pectate lyase), PL3_2 (pectate lyase) and PL4_1 (rhamnogalacturonan endolyase) (Supplementary Table S6). Approximately 73% of all identified polysaccharide lyases are predicted to be secreted (Table 4).

**Figure 4:**
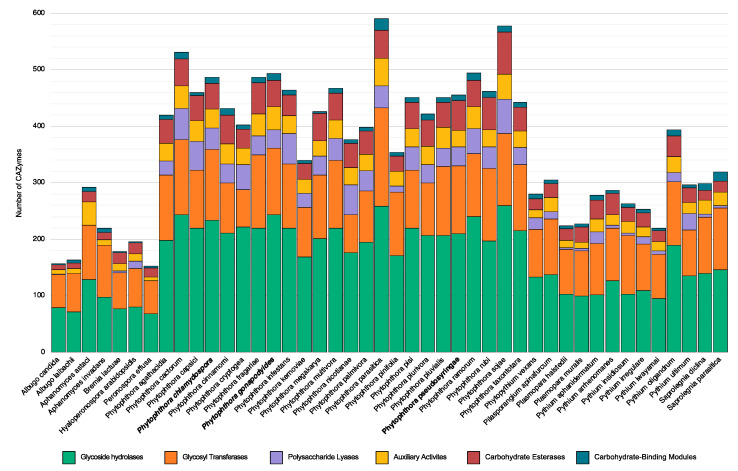
Distribution of CAZymes across 44 oomycete genomes. The arsenal of carbohydrate active enzymes in *Phytophthora* species is larger than what is observed in other oomycete species. In particular the difference in glycoside hydrolase repertoire is noticeable.

We extended this analysis by comparing the CAZyme repertoires of 44 oomycete species with different host ranges and broad lifestyles (Figure 4 and Supplementary Table S1). In agreement with previous studies [119], our results show that *Phytophthora* species tend to have larger numbers of CAZymes compared to other oomycete taxa (Figure 4). Specifically examining 24 *Phytophthora* species, the average number of CAZymes is 450. On average, each *Phytophthora* genome encodes 215 glycoside hydrolases, 111 glycoside transferases, 38 polysaccharide lyases, 34 proteins involved in auxiliary activities, 46 carbohydrate esterases and 9 proteins with carbohydrate-binding modules. The number of CAZymes identified in *Ph. pseudosyringae* is close to the average *Phytophthora* (Figure 4). The two clade 6 aquatic *Phytophthora* species have an expanded repertoire of CAZymes relative to the average *Phytophthora*, with 483 CAZymes in *Ph. chlamydospora* and 487 CAZymes for *Ph. gonapodyides* (Figure 4). In particular, they have a higher than average number of glycoside hydrolases, with 234 in *Ph. chlamydospora* and 243 in *Ph. gonapodyides* (Figure 4 and Table 4). All three genomes have a higher than average number of glycoside transferases, with 125 in *Ph. chlamydospora*, 118 in *Ph. gonapodyides* and 120 in *Ph. pseudosyringae* (Figure 4 and Table 4). *Ph. gonapodyides* also has a higher than average number of proteins involved in auxiliary activities with 41 proteins (Figure 4 and Table 4).

**Figure 5:**
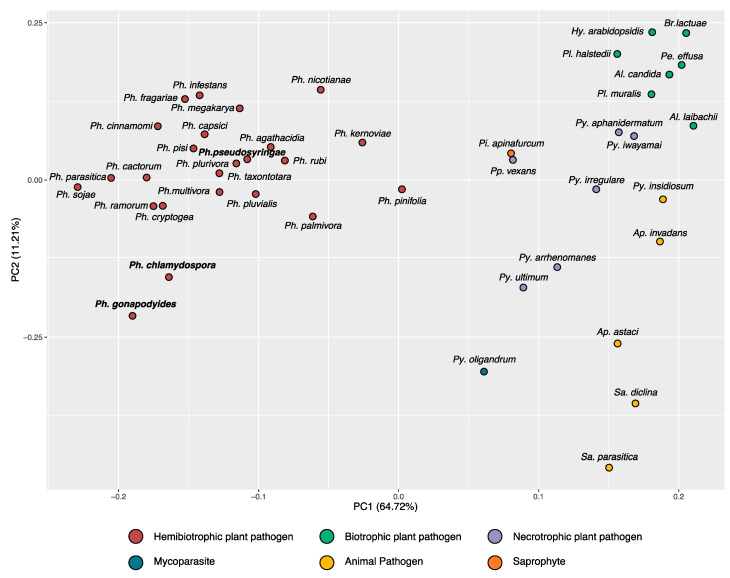
PCA clustering of oomycete species based on copy numbers of glycoside hydrolase families. Genus abbreviations are as follows: *Albugo* (*Al*.), *Aphanomyces* (*Ap*.), *Bremia* (*Br*.), *Hyaloperonospora* (*Hy*.), *Peronospora* (*Pe*.), *Phytophthora* (*Ph*.), *Phytopythium* (*Pp*.), *Pilasporangium* (*Pi*.), *Plasmopara* (*Pl*.), *Pythium* (*Py*.), *Saprolegnia* (*Sp*.). Colored dots relate to lifestyle. Species with similar lifestyles are clustered together suggesting oomycete lifestyles may be linked to their glycoside hydrolase repertoires.

Interestingly, principal component analysis (PCA) of glycoside hydrolase family copy numbers clusters species with similar lifestyles together (Figure 5). All downy mildew species (*Albugo*, *Bremia*, *Hyaloperonospora*, *Peronospora*, and *Plasmopara*) cluster tightly together (Figure 5), despite evolving obligate biotrophism independently (Figure 2) [96,120]. Plant pathogenic *Pythium* species are also more distantly clustered (Figure 5). The mycoparasite *Pythium oligandrum* is placed distantly to all other *Pythium* species (Figure 5), suggesting it may have a specialised repertoire of glycoside hydrolases involved in infection of fungi and oomycetes [121]. The animal pathogens Aphanomyces astaci, *Saprolegnia diclina* and *Saprolegnia parasitica* are clustered together (Figure 5). Aphanomyces invadans is clustered with the mammalian pathogen Pythium insidiosum (Figure 5). The intermediate genera, *Pilasporangium* and *Phytopythium*, are clustered together between *Phytophthora* and *Pythium* (Figure 5). All *Phytophthora* species are clustered together, over a wide area (Figure 5). *Phytophthora chlamydospora* and *Ph. gonapodyides* are clustered together but relatively distant to all other *Phytophthora* species (Figure 5). This suggests that these opportunistic aquatic *Phytophthora* species may have distinctive glycoside hydrolase arsenals. Furthermore, they are placed distantly from their closest relative in the dataset *Ph. pinifolia* (Figure 2 and Figure 5). Examining individual glycoside hydrolase families, both *Ph. chlamydospora* and *Ph. gonapodyides* have expansions of glycoside hydrolase families 1, 3, 5, 10, 13 and 43 (Supplementary Figure S2). These results suggest that oomycete lifestyles may be linked to their CAZyme repertoires, in particular glycoside hydrolase families. These findings are similar to recent analyses showing clustering of oomycete species with similar lifestyles based on metabolic networks [122,123].

### 3.6. Tandemly Duplicated Genes

Tandemly duplicated genes are duplicated genes that are located adjacent to each other in the genome. Analysis of tandemly duplicated genes using BLASTp led to the identification of 2513 (14.1%) tandemly duplicated genes in *Ph. chlamydospora*, 1863 (8.0%) in *Ph gonapodyides* and 2225 (12.8%) in *Ph. pseudosyringae* (Table 5). The tandemly duplicated genes are located in 833 to 979 tandem clusters (Table 5 and Supplementary Table S7). On average, each cluster has between 2.24 and 2.57 tandemly duplicated genes (Table 5). Fewer tandemly duplicated genes were identified in *Ph. gonapodyides* compared to *Ph. chlamydospora* and *Ph. pseudosyringae* (Table 5)*,* however, this analysis may have been limited by the poor assembly contiguity of *Ph. gonapodyides*. Overall, counts of tandemly duplicated genes are similar to those observed in other *Phytophthora* genomes [124]. Proteins predicted to be secreted are significantly overrepresented in tandemly duplicated clusters (*p* < 0.05 (Fisher’s exact test)), with 354 *Ph. chlamydospora* secreted proteins found in tandem clusters, 265 from *Ph. gonapodyides* and 328 from *Ph. pseudosyringae* (Table 5). This adds further evidence that tandem gene duplication has played a role in the expansion of oomycete secretomes [124]. Our results show that putative effector proteins are numerous in tandem clusters. For example, in *Ph. chlamydospora*, 33 elicitins out of a total of 57 (58%) are located in 12 tandem clusters (Supplementary Table S7). Clusters of elicitins genes have also been reported in other *Phytophthora* species [6,105]. Similarly, 20 out of a total 34 CAP proteins (59%) are found in eight tandem clusters (Supplementary Table S7). Tandem gene duplication has also played a role in the expansion of *Phytophthora* CAZyme arsenals. For example, in *Ph. chlamydospora* 175 out of a total of 483 proteins (36%) annotated as putative CAZymes (Table 4**)** are found in tandem clusters. We observed similar trends in all three genome assemblies.

### 3.7. LC-MS/MS Characterisation of Phytophthora Extracellular Proteomes

Here we used a mass spectrometry-based approach to characterise the in vivo secretomes and extracellular proteomes of *Ph. chlamydospora*, *Ph. gonapodyides* and *Ph. pseudosyringae*. Each species was cultured under two conditions with different media types −10% V8 juice or 10% cV8 juice. Extracellular medium was harvested 10 days after inoculation. To minimise the possibility of hyphal lysis, extracellular medium was carefully harvested using a syringe without disrupting the *Phytophthora* hyphae. Proteins were extracted from extracellular medium and subjected to LC-MS/MS to identify extracellular proteins. Proteins were identified by searching spectra against the predicted proteome of each species. Protein groups (a group of indistinguishable proteins based on identified peptides) were considered present in a condition if they were identified based on at least two peptides and present in at least two out of three independent biological replicates. Protein groups were considered unique to a condition if they were not detected in any replicate of other conditions.

**Figure 6:**
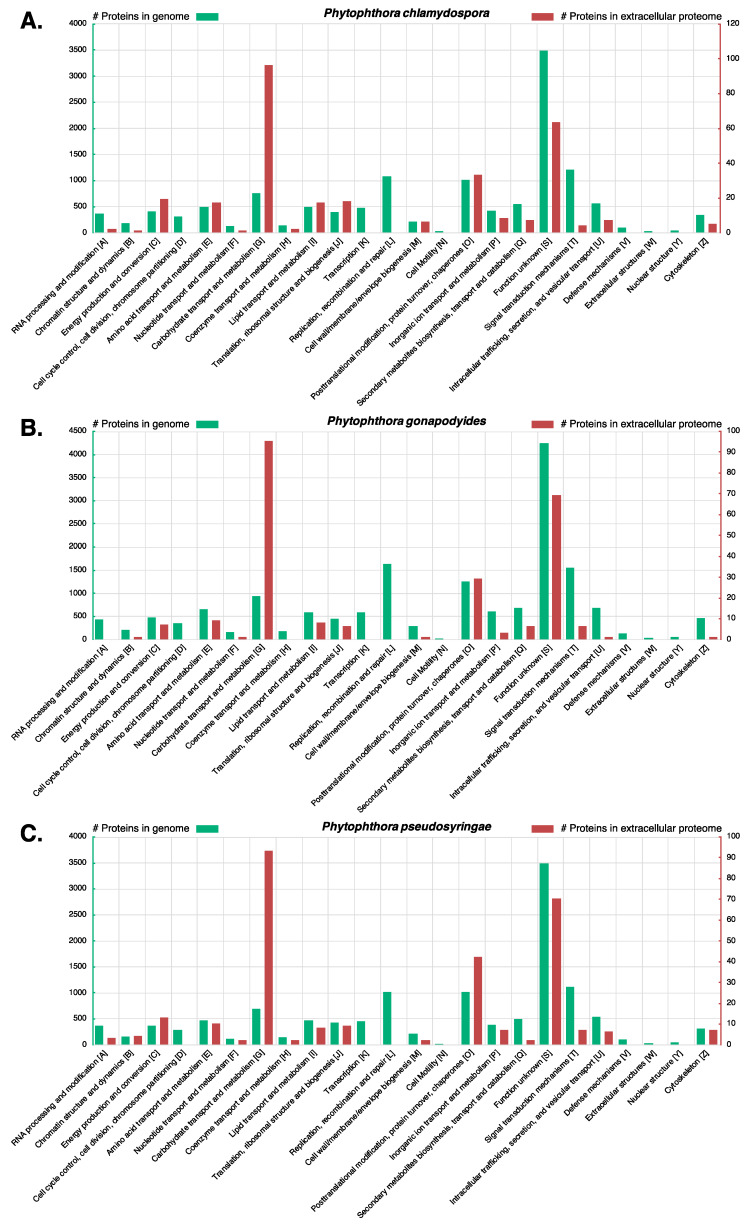
eggNOG functional annotation of the genome and LC-MS/MS extracellular proteome of (**A**) *Phytophthora chlamydospora*, (**B**) *Phytophthora gonapodyides* and (**C**) *Phytophthora pseudosyringae*. Note that counts for extracellular proteins are shown on a secondary axis (right) with a different scale.

Examining *Ph. chlamydospora*, 302 protein groups (327 proteins) were identified in the cV8 samples and 251 protein groups (274 proteins) were identified in the V8 samples (Supplementary Table S8). 20 protein groups (20 proteins) were unique to the cV8 samples and 14 protein groups (17 proteins) were unique to the V8 samples (Supplementary Table S8). In total across the two conditions, 321 protein groups (351 proteins) were identified (Table 6 and Supplementary Table S8), of which 149 proteins (42%) overlap with the predicted secretome of *Ph. chlamydospora*. Reducing the strictness of the SignalP 3 analysis by considering all positive HMM predictions without applying additional constraints, 196 proteins (56%) are predicted to be secreted. This compares favourably to *Ph. plurivora*, where 60% of extracellular proteins identified by LC-MS/MS contained predicted N-terminal signal peptides [16]. The proteins lacking signal peptides may be present in the extracellular medium due to contamination of intracellular proteins caused by hyphal lysis during protein extraction. Additionally, these proteins may have legitimate signal peptides that cannot be detected due to inaccurate gene annotation, i.e., gene models with a truncated N-terminus will lack N-terminal signal peptides [15]. It is also possible that they are leaderless secretory proteins (LSPs), lacking signal peptides that enter non-classical secretory pathways. Extracellular proteins that lack signal peptides were submitted to SecretomeP 2.0, an ab initio predictor of non-classically secreted proteins [125]. A total 78 proteins (22.2%) had a SecretomeP NN-score greater than 0.5, suggesting that they may be non-classically secreted proteins. It is important to note that SecretomeP has only been trained on mammalian LSPs, therefore its accuracy at predicting oomycete LSPs is unclear. eggNOG assigned 298 extracellular proteins (84.9%) to one or more functional COG groups. The most numerous functional categories were carbohydrate transport and metabolism (96 proteins, 32.2%), function unknown (63 proteins, 21.1%), posttranslational modification, protein turnover and chaperones (33 proteins, 11.1%), energy production and conversion (19 proteins, 6.4%), translation, ribosomal structure and biogenesis (18 proteins, 6.0%), amino acid transport and metabolism (17 proteins, 5.7%), and lipid transport and metabolism (17 proteins, 5.7%) (Figure 6A). The high number of extracellular proteins involved in transport and metabolism is to be expected for osmotrophs which obtain their nutrients externally from the environment or from their hosts. The proportion of extracellular proteins classified as carbohydrate transport and metabolism (96 proteins; 32.2%) is particularly enriched relative to the total genome (746 proteins; 6.2%) (Figure 6A). These proteins may be involved in the breakdown of host plant cells as well in the acquisition and uptake of nutrients. No extracellular proteins (for any species) were annotated as being involved in “cell cycle control, cell division, chromosome partitioning”, “transcription”, “replication, recombination and repair”, “cell motility”, “defense mechanisms”, “extracellular structures” or “ nuclear structure” (Figure 6A–C). This suggests that hyphal lysis is unlikely to have occurred during protein extraction as these annotations are associated with intracellular processes or cell structures. A large number of known effector families were also detected, including proteins with PAN/Apple domains (10), transglutaminase elicitors (6), elicitins (5), proteins belonging to the cysteine-rich secretory protein family (4), and NLPs (2) (Table 6). An extracellular berberine-like protein was also detected (Table 6). Berberine-like proteins were previously reported as putative virulence factors in *Ph. infestans* [69] and are thought to be involved in infection by the biosynthesis of alkaloids and the production of reactive oxygen species. Berberine-like proteins were also detected in the extracellular proteomes of *Ph. infestans* and *Ph. plurivora* [15,16,69]. Additionally, an extracellular ribonuclease was detected (Table 6). Secreted ribonucleases have been reported as effectors in the fungal plant pathogen *Blumeria graminis* [126]. Ribonucleases were also detected in the *Ph. infestans* extracellular proteome [15]. In total, 140 extracellular proteins (40%) have homologs in PHI-Base (Table 6). A large number of extracellular CAZymes were also identified including glycoside hydrolases (60), polysaccharide lyases (7), carbohydrate esterases (4), auxiliary activities (8) and proteins with carbohydrate-binding modules (3) (Table 6).

Examining *Ph. gonapodyides*, 237 protein groups (268 proteins) were identified in the cV8 samples and 196 protein groups (230 proteins) were identified in the V8 samples (Supplementary Table S8). 17 protein groups (19 proteins) were unique to the cV8 samples and 9 protein groups (15 proteins) were unique to the V8 samples (Supplementary Table S8). In total across the two conditions, 246 protein groups (283 proteins) were identified (Table 6 and Supplementary Table S8), of which 133 proteins (47%) overlap with the predicted secretome of *Ph. gonapodyides*. 167 proteins (59%) have positive SignalP 3 HMM predictions, ignoring additional cut-offs. An additional 76 proteins (26.9%) have a positive prediction from SecretomeP, suggesting non-classical secretion. Functional annotation using eggNOG assigned 238 extracellular proteins (84.1%) to one or more COG categories. Overall the functional profile was similar to that of *Ph. chlamydospora*, with the most numerous functional categories being carbohydrate transport and metabolism (95 proteins, 39.9%), function unknown (69 proteins, 29.0%), posttranslational modification, protein turnover, chaperones (29 proteins, 12.2%), amino acid transport and metabolism (9 proteins, 3.8%), lipid transport and metabolism (8 proteins, 3.4%) and energy production and conversion (7 proteins, 2.9%) (Figure 6B). Identified effector families include proteins with PAN/Apple domains (8), transglutaminase elicitors (6), elicitins (8), members of the cysteine-rich secretory protein family (4), NLPs (4), a berberine-like protein and a ribonuclease (Table 6). Some 96 (34%) extracellular proteins have homologs in PHI-Base (Table 6). A similar number of extracellular CAZymes were detected including glycoside hydrolases (68), polysaccharide lyases (9), carbohydrate esterases (4), auxiliary activities (5) and proteins with carbohydrate-binding modules (4) (Table 6).

Examining *Ph. pseudosyringae,* 280 protein groups (296 proteins) were identified in the cV8 samples and 247 protein groups (259 proteins) were identified in the V8 samples (Supplementary Table S8). 18 protein groups (22 proteins) were unique to the cV8 samples and 6 protein groups (6 proteins) were unique to the V8 samples (Supplementary Table S8). In total across the two conditions, 313 protein groups (331 proteins) were identified (Table 6 and Supplementary Table S8), of which 145 proteins (44%) overlap with the predicted secretome of *Ph. pseudosyringae*. 188 proteins (56.8%) proteins have positive SignalP 3 HMM predictions without applying cut offs. An additional 67 proteins (20.2%) have a positive prediction from SecretomeP. eggNOG functional annotation assigned 279 extracellular proteins (84.3%) to one or more COG functional categories. The high-level functional annotation of the *Ph. pseudosyringae* is similar to that of *Ph. chlamydospora* and *Ph. gonapodyides* (Figure 6C). The most numerous functional categories are carbohydrate transport and metabolism (93 proteins, 33.3%), function unknown (70 proteins, 25.1%), posttranslational modification, protein turnover, chaperones (42 proteins, 15.1%), energy production and conversion (13 proteins, 4.7%), amino acid transport and metabolism (10 proteins, 3.6%), translation, ribosomal structure and biogenesis (9 proteins, 3.2%) and lipid transport and metabolism (8 proteins, 2.9%) (Figure 6C). The number of effector families is also similar and includes proteins with PAN/Apple domains (5), transglutaminase elicitors (8), elicitins (4), members of the cysteine-rich secretory protein family (4), NLPs (4) and ribonucleases (3) (Table 6). We also detected an extracellular PcF phytotoxin from *Ph. pseudosyringae*, which was absent in the extracellular proteomes of both *Ph. chlamydospora* and *Ph. gonapodyides* (Table 6). Unlike *Ph. chlamydospora* and *Ph. gonapodyides*, we did not identify any berberine-like extracellular proteins from *Ph. pseudosyringae*, although its predicted secretome encodes a copy (Table 3). Some 109 extracellular proteins (33%) have homologs in PHI-Base (Table 6). Overall the CAZyme content of the *Ph. pseudosyringae* extracellular proteome is also similar with glycoside hydrolases (61), polysaccharide lyases (5), carbohydrate esterases (2), auxiliary activities (4) and proteins with carbohydrate-binding modules (3) (Table 6).

Very few cytoplasmic effectors were detected in our analyses of all three species. CRNs were absent from the extracellular proteomes of all three species. No putative RxLRs were identified in the extracellular proteome of *Ph. chlamydospora* or *Ph. gonapodyides*. Only two putative RxLRs were identified in the extracellular proteome of *Ph. pseudosyringae* PHPS_09091 and PHPS_15662. PHPS_09091 was detected in all replicates of both V8 and cV8 media with 4 unique peptides (Supplementary Table S8) and was identified as an RxLR based on the Win, Regex and HMM methods (Supplementary Table S5). Similarly, PHPS_15662 was identified in all replicates of both V8 and cV8 media with 4 unique peptides (Supplementary Table S8). PHPS_15662 was identified only using the homology method and does not contain a RxLR-like motif (Supplementary Table S5), therefore it is not likely to be a legitimate RxLR effector. It is not surprising that so few cytoplasmic effectors were identified as it is possible that most cytoplasmic effectors are secreted from haustoria [127,128]. However, Meijer et al. (2014) report the detection of several *Ph. infestans* RxLRs and CRNs being released from hyphae in the absence of haustoria.

Previously, LC-MS/MS analysis of *Ph. infestans* identified 31 extracellular proteins that contained a single transmembrane domain [15]. These proteins are thought to be membrane proteins that are found in the extracellular medium due to proteolytic ectodomain shedding by sheddases. We also detected a large number of extracellular proteins that contain a single transmembrane domain. This included 33 proteins in *Ph. chlamydospora*, 25 in *Ph. gonapodyides* and 29 in *Ph. pseudosyringae* (Table 6). Of these, 17, 14 and 17 are homologous to the 31 *Ph. infestans* proteins. Similar to what was observed in *Ph. infestans*, the majority of identified transmembrane domains are found in the protein C-terminus.

Interestingly, we detected a number of extracellular proteins with KDEL or KDEL-like (HDEL or SDEL) C-terminal motifs. These are endoplasmic retention (ER) motifs that are usually associated with preventing protein secretion, signalling proteins to be retained in the ER lumen [129]. Proteins with KDEL motifs are usually excluded from in silico secretome studies. In *Ph. chlamydospora* we identified three such proteins PHCH_06832, PHCH_07252, and PHCH_15931. Both PHCH_06832 and PHCH_07252 are paralogs belonging to the same protein group. They were identified in all replicates of both V8 and cV8 media with four unique peptides (Supplementary Table S8). Both proteins contain a C-terminal HDEL motif and were annotated as belonging to heat shock protein (Hsp) 70 family. PHCH_15931 was identified in a total of five out of six replicates across the two conditions with five unique peptides and has a C-terminal KDEL motif (Supplementary Table S8). It was annotated as a calreticulin, which is an ER associated calcium-binding protein [130]. In *Ph. gonapodyides*, only one such protein was identified, PHGO_06390, which was detected in two out of three replicates of the cV8 samples and has a C-terminal SDEL motif (Supplementary Table S8). In *Ph. pseudosyringae*, three proteins were identified PHPS_03476, PHPS_04861, and PHPS_06172. PHPS_04861 is orthologous to PHGO_06390, has a C-terminal SDEL motif and was identified in all replicates of both conditions with 3 unique peptides (Supplementary Table S8). PHPS_03476 has a C-terminal KDEL motif and is orthologous to PHCH_15931 and was identified in all replicates of both conditions, with a total of seven unique peptides (Supplementary Table S8). PHPS_06172, a Hsp90 protein, contains a C-terminal KDEL motif and was identified in a total of four replicates across the two conditions, with two peptides, only one of which was unique (Supplementary Table S8). Inspecting the *Ph. infestans* extracellular proteins identified by Meijer et al. (2014)[15], there were six extracellular proteins identified that contain C-terminal KDEL/HDEL/SDEL motifs, five of which are orthologous to those identified above. As we detected these KDEL/KDEL-like motif-containing proteins in the extracellular medium, it suggests that their ER retention motifs are masked or perhaps they escape ER retrieval due to saturation of KDEL receptors [130].

### 3.8. LC-MS/MS Identification of Mycelial Proteins

We used mass-spectrometry to characterise the mycelial proteomes of *Ph. chlamydospora*, *Ph. gonapodyides* and *Ph. pseudosyringae*, and to understand how they change in response to oxidative stress and high temperatures. Proteins were extracted from mycelia grown under three conditions: “normal”—mycelia grown for 10 days at optimum temperatures—“heat”—mycelia grown for 7 days at optimum temperatures then switched to 30 °C for 3 days—and “oxidative stress”—mycelia grown for 10 days at optimum temperatures followed by exposure to 1 mM H_2_O_2_ for 3 h. 

Examining *Ph. chlamydospora*, a total of 2592 protein groups (2635 proteins) were identified across the three conditions (Supplementary Table S9). Under the normal condition, 2418 protein groups (2461 proteins) were identified (Supplementary Table S9). 123 protein groups (130 proteins) were uniquely detected under the normal conditions (Supplementary Table S9) and were significantly enriched for oxidoreductase activity (GO:0016491). Only three protein groups (three proteins) were uniquely detected in the heat-treated samples. These included a protein with an FHA domain (PHCH_03368) and a histidine phosphatase (PHCH_17500) (Supplementary Table S9). Only 5 protein groups (5 proteins) were uniquely detected in the oxidative stress samples (Supplementary Table S9). These included a CAF1 ribonuclease (PHCH_05536), an integrator complex subunit (PHCH_09875), a protein kinase (PHCH_13661), a DNA photolyase (PHCH_14550) and a methyltransferase (PHCH_16784) (Supplementary Table S9). 328 (12.4%) of all identified proteins have one or more predicted transmembrane helices. Furthermore, 131 (5.0%) of all identified proteins belong to the predicted secretome, while 213 (8.1%) proteins were also were also identified in the extracellular proteome. Amongst the identified proteins were several effector families, including NLPs (1), transglutaminase elicitors (3), elicitins (5), PAN domain containing proteins (5), CAP family proteins (5), RxLRs (6), and CRNs (14) (Supplementary Table S9). Additionally, 81 CAZymes were identified from *Ph. chlamydospora* mycelium (Supplementary Table S9).

Examining *Ph. gonapodyides*, a total of 2745 protein groups (2,840 proteins) were identified across the three conditions (Supplementary Table S9). Under the normal condition, 2360 protein groups (2436 proteins) were detected (Supplementary Table S9). Only one protein was uniquely detected in this condition, a member of glycoside hydrolase family 31 (PHGO_11274). Under heat treatment 12 protein groups (12 proteins) were uniquely detected, 3 of these contain predicted transmembrane helices and 2 are predicted to be secreted. Included amongst these proteins, were an acetyltransferase (PHGO_14090), an ABC transporter (PHGO_22460), an auto-transporter adhesin (PHGO_20954), a metallopeptidase (PHGO_13577), a starch-binding protein (PHGO_10964), an exoribonuclease (PHGO_00249), a Maf like protein (PHGO_22144) and an Arf GTPase activating protein (PHGO_17826) (Supplementary Table S9). In response to oxidative stress, 34 protein groups (37 proteins) were uniquely detected. Amongst these, 12 proteins (32.4%) have one or more predicted transmembrane helices and three belong to the predicted secretome. These proteins include two peroxidases (PHGO_01245 and PHGO_22132), both of which are predicted to be secreted. Similar to *Ph. chlamydospora*, 346 (12.2%) of all identified *Ph. gonapodyides* mycelial proteins have one or more predicted transmembrane helices. Furthermore, 131 (4.6%) of identified mycelial proteins also belong to the predicted secretome and 144 (5.1%) were also identified in the extracellular proteome (Supplementary Table S9). We also detected a number of effector families, including CRNs (2), RxLRs (2), transglutaminase elicitors (4), PAN domain-containing proteins (5), and elicitins (7) (Supplementary Table S9). Additionally, 89 CAZymes were identified from *Ph. gonapodyides* mycelium (Supplementary Table S9).

Examining *Ph. pseudosyringae*, a total of 3195 protein groups (3245 proteins) were identified across the three conditions (Supplementary Table S9). Under the normal condition, 2223 protein groups (2248 proteins) were detected, 22 protein groups (22 proteins) of which were uniquely detected in this condition (Supplementary Table S9). 32 protein groups (33 proteins) were uniquely detected in the heat-treated samples which included three proteins predicted to be secreted and four proteins with predicted transmembrane helices. Heat-treated samples were significantly enriched for chaperone binding (GO:0051087). We detected 103 unique protein groups (106 proteins) in response to H_2_O_2_ treatment, including three proteins predicted to be secreted and 30 proteins with at least one predicted transmembrane helix. Of the proteins identified across all conditions, 445 (13.7%) of mycelial proteins contain one or more predicted transmembrane helices. Furthermore, 160 (4.9%) belong to the predicted secretome, and 204 (6.3%) were also identified in the extracellular proteome (Supplementary Table S9). We also detected effector families, including NLPs (2), CAP family proteins (3), elicitins (4), transglutaminase elicitors (5), PAN domain-containing proteins (5), RxLRs (8) and CRNs (18) (Supplementary Table S9). Additionally, 103 CAZymes were identified from *Ph. pseudosyringae* mycelium (Supplementary Table S9).

**Figure 7:**
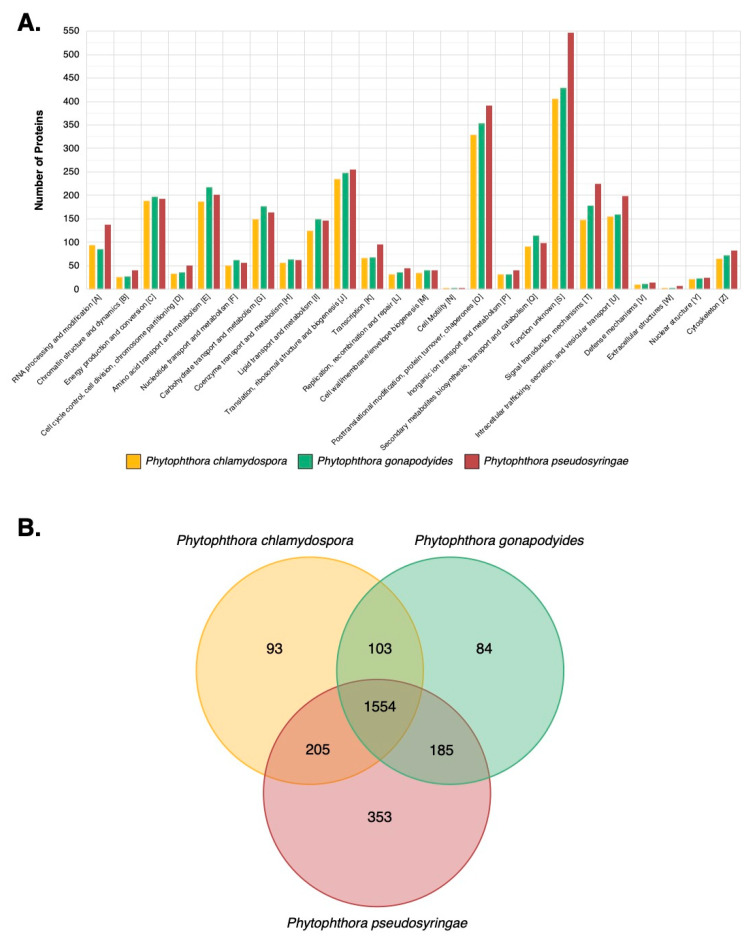
Functional annotation of identified mycelial proteins. (**A**) Identified mycelial proteins were functionally annotated using eggNOG-Mapper. (**B**) Venn diagram showing the number of identified mycelial protein families shared between each species.

Overall, the functional annotation of all identified mycelial proteins is similar between each of the three species (Figure 7A). Clustering with MCL grouped identified mycelial proteins from the three species into 2577 protein families, of which 1554 families were shared by all three species (Figure 7B). More proteins were common between *Ph. chlamydospora* and *Ph. pseudosyringae* (205) and between *Ph. gonapodyides* and *Ph. pseudosyringae* (185) than between *Ph. chlamydospora* and *Ph. gonapodyides* (103) (Figure 7B). 93 mycelial protein families were unique to *Ph. chlamydospora*, 84 to *Ph. gonapodyides* and 353 to *Ph. pseudosyringae* (Figure 7B), indicating increased variation across *Phytophthora* clades.

### 3.9. Phylostratigraphy Analysis

Taxonomically restricted genes were identified using phylostratigraphy. Homologs were identified for each *Phytophthora* protein-coding gene by performing BLAST searches against a large protein database (18,084,866 proteins) with broad phyletic distribution [85]. Genes were assigned to one of seven phylostrata (cellular organisms, eukaryotes, Stramenopiles, oomycetes, Peronosporales, *Phytophthora* or species-specific orphans) based on their conservation in other taxonomic lineages.

**Figure 8:**
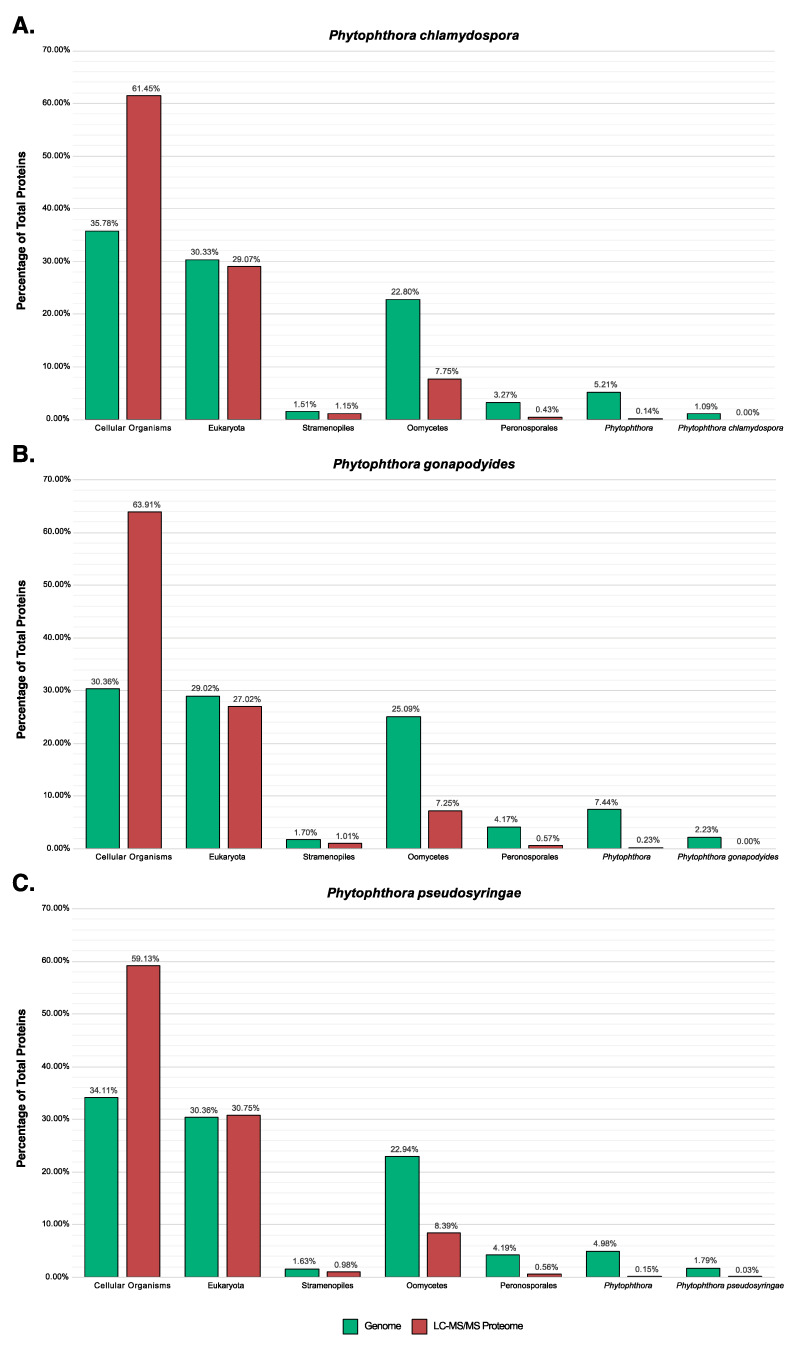
Phylostratigraphy analysis of *Ph. chlamydospora* (**A**), *Ph. gonapodyides* (**B**), and *Ph. pseudosyringae* (**C**), showing the proportion of genes assigned to each phylostratum for both the genome and the LC-MS/MS proteome.

Overall the proportion of genes assigned to each phylostrata are similar between the three *Phytophthora* genomes (Figure 8). On average, 33.4% of genes were assigned to the phylostratum cellular organisms (i.e., homologs are present in eukaryotes and prokaryotes), 29.9% are unique to eukaryotes, 1.6% are unique to stramenopiles, 23.6% are unique to oomycetes, 3.9% are unique to Peronosporales, 5.9% are unique to *Phytophthora* and 1.7% are orphans unique to one *Phytophthora* species (Figure 8). Individually, 194 orphans (1.1%) were identified for *Ph. chlamydospora*, 520 (2.2%) for *Ph. gonapodyides* and 312 (1.8%) for *Ph. pseudosyringae* (Figure 8). *Ph. gonapodyides* had a smaller proportion of genes identified as originating in cellular organisms (30.4%), compared to *Ph. chlamydospora* (35.8%) and *Ph. pseudosyringae* (34.1%) (Figure 8B). In addition, *Ph. gonapodyides* had a higher proportion of genes identified as being unique to oomycetes (25.1%), unique to *Phytophthora* (7.4%) and species-specific (2.2%) (Figure 8B). This suggests that the increased gene repertoire of *Ph. gonapodyides* is due to expansions of more recently evolved gene families, i.e., genes unique to oomycetes, *Phytophthora*, and *Ph. gonapodyides*, as opposed to expansions of more ancient genes that are conserved in other eukaryotes or prokaryotes. Overall the proportion of genes per phylostratum is similar to previous phylostratigraphic analyses for other *Phytophthora* genomes [124].

We coupled our phylostratigraphy analysis with the mass spectrometry data. Compared to the overall genome, a much larger proportion (approximately 61.5%) of identified proteins belongs to the phylostratum “cellular organisms” (Figure 8). This suggests that the majority of identified proteins are evolutionarily conserved proteins that possibly play roles in conserved housekeeping functions. Only 8.1% to 9.1% of identified proteins belong to the phylostratum oomycetes or “younger” (Figure 8). Furthermore, only 0.57% to 0.80% of identified proteins belong to the phylostratum Peronosporales or “younger” (Figure 8). This suggests that the more recently evolved genes may be under tighter transcriptional control or expressed only in specific scenarios. Additionally, some of the proteins identified as being species-specific may not be legitimate genes.

## 4. Conclusions

Here, we have sequenced the genomes for three ubiquitous *Phytophthora* species—*Ph. chlamydospora*, *Ph. gonapodyides* and *Ph. pseudosyringae*. Using bioinformatics methods, comparative genomics, and mass spectrometry, we provide a comprehensive characterization of the nuclear genomes, mitochondrial genomes, in silico secretomes, extracellular proteomes, and mycelial proteomes of each species. These genome resources will be useful for future studies to understand the lifestyles of these widespread *Phytophthora* species.

## Figures and Tables

**Table 1:** Details of the isolates used in this study.

Species	Isolate Number	Source	Date Collected	Location (Latitude, Longitude)	BioProject Accession
*Phytophthora chlamydospora*	P17-99	*Rhododendron ponticum* leaf baiting of a stream	08/08/2017	Tollymore forest, Co. Down, Northern Ireland, UK (54°13’16.9”N, 5°55’49.3”W)	PRJNA599565
*Phytophthora gonapodyides*	P17-128	*Rhododendron ponticum* leaf baiting of a stream	28/08/2017	Rostrevor forest, Co. Louth, Ireland (54°07’43.4”N, 6°09’24.2”W)	PRJNA599567
*Phytophthora pseudosyringae*	PR13-731	Bleeding bark canker of *Fagus sylvatica*	31/07/2015	Mullaghreelan forest, Co Kildare, Ireland (52°56’00.4”N, 6°52’41.0”W)	PRJNA599564

**Table 2:** Genome assembly statistics.

Genome Assembly	*Phytophthora chlamydospora*	*Phytophthora gonapodyides*	*Phytophthora pseudosyringae*
Estimated Genome Size (bp)	51,100,498	65,211,327	51,026,880
Assembly Size (bp)	45,264,984	61,088,431	47,882,184
Number of Scaffolds	4077	16,449	3627
N50 (bp)	26,559	5455	26,492
L50	466	2927	526
GC Content	55.7%	55.7%	54.8%
Sequencing Coverage	98x	76x	102x
Repeat Masked	9.0%	16.1%	13.3%
Estimated Heterozygosity	0.68%	1.88%	0.15%
BUSCO Completeness	97.8%	87.2%	94.1%
**Gene Prediction**			
Gene Models	17,872	23,348	17,439
CDS density	56.3%	43.3%	49.2%
BUSCO Completeness	97.5%	88.1%	94.4%
Proteins with Pfam domains	10,759 (60.2%)	12,181 (52.2%)	10,130 (58.1%)

**Table 3:** Counts of putative effector proteins.

	*Phytophthora chlamydospora*	*Phytophthora gonapodyides*	*Phytophthora pseudosyringae*
Secreted Proteins	1140 (6.38%)	1291 (5.53%)	1131 (6.49%)
ApoplastP Hits	554 (48.5%)	630 (48.8%)	533 (47.1%)
PHI-Base homologs	249 (21.8%)	234 (18.1%)	243 (21.5%)
**Apoplastic effectors**			
Berberine-like proteins	1 (1)	2 (1)	3 (1)
Cysteine-rich secretory proteins (CAP)	34 (22)	34 (25)	31 (22)
Elicitins	57 (45)	59 (47)	45 (34)
Necrosis-inducing proteins	25 (19)	33 (22)	22 (19)
PAN/Apple domain	25 (21)	32 (20)	20 (15)
PcF phytotoxins	1 (0)	1 (1)	1 (1)
Transglutaminase elicitors	14 (11)	16 (11)	17 (11)
**Proteases and protease inhibitors**			
Aspartyl proteases	59 (4)	53 (3)	26 (2)
Papain family cysteine proteases	19 (8)	22 (6)	20 (8)
Serine proteases	13 (6)	12 (4)	18 (9)
Kazal-type protease inhibitors	13 (12)	15 (11)	16 (10)
Cathepsin propeptide inhibitors	4 (2)	4 (1)	4 (3)
**Cytoplasmic effectors**			
Crinklers	77 (28)	80 (18)	90 (37)
RxLRs	132 (132)	132 (132)	186 (186)
**Polysaccharide modifying enzymes**			
Cellulases	30 (7)	35 (4)	22 (5)
Lytic polysaccharide mono-oxygenases	6 (5)	4 (2)	5 (5)
Cutinases	3 (2)	4 (2)	5 (5)
Chitinases	1 (1)	2 (0)	2 (1)
Fungal cellulose binding domains	8 (6)	10 (7)	8 (8)
Pectate lyases	34 (23)	25 (14)	31 (23)
Pectin acetylesterases	6 (4)	6 (3)	6 (5)
Pectinesterases	7 (6)	7 (5)	15 (9)

Putative effectors were annotated based on Pfam domains or manually curated (CRNs and RxLRs). Numbers in brackets represent proteins belonging to the predicted secretome.

**Table 4:** Counts of carbohydrate-active enzymes (CAZymes) in *Phytophthora* genomes.

	*Phytophthora chlamydospora*	*Phytophthora gonapodyides*	*Phytophthora pseudosyringae*
Glycoside Hydrolases	234 (144)	243 (123)	210 (121)
Glycosyl Transferases	125 (11)	118 (10)	120 (12)
Polysaccharide Lyases	38 (27)	33 (22)	33 (27)
Carbohydrate Esterases	46 (16)	46 (12)	53 (22)
Auxiliary Activities	33 (14)	41 (11)	29 (11)
Carbohydrate-Binding Modules	10 (2)	12 (4)	10 (1)
Total CAZymes	483 (213)	487 (179)	453 (194)

Numbers in brackets represent proteins belonging to the predicted secretome. Full list of annotations per gene is available in Supplementary Table S6.

**Table 5:** Tandem gene duplication.

	*Phytophthora chlamydospora*	*Phytophthora gonapodyides*	*Phytophthora pseudosyringae*
Tandem Clusters	979	833	874
Genes in Tandem Clusters	2513 (14.1%)	1863 (8.0%)	2225 (12.8%)
Average Number of Genes Per Tandem Cluster	2.57	2.24	2.55
Secreted Proteins in Tandem Clusters	354 (31.1%)	265 (20.5%)	328 (29.0%)

**Table 6:** Extracellular *Phytophthora* proteins identified by LC-MS/MS that are putatively involved in osmotrophy or virulence.

	*Phytophthora chlamydospora*	*Phytophthora gonapodyides*	*Phytophthora pseudosyringae*
Total protein groups identified	321	246	313
Total proteins identified	351	283	331
PAN/Apple domain	10	8	5
Transglutaminase elicitor	6	6	8
Elicitin	5	8	4
Cysteine-rich secretory protein family (CAP)	4	4	4
Necrosis inducing protein	2	4	2
PcF phytotoxin	0	0	1
Ribonuclease	1	1	3
Berberine-like protein	1	1	0
Glycoside hydrolases	60	68	61
Polysaccharide lyases	7	9	5
Carbohydrate esterases	4	4	2
Auxiliary activities	8	5	4
Carbohydrate-binding modules	3	4	3
PHI-Base homologs	140	96	109
Apoplastic proteins	99	90	92
Single transmembrane proteins	33	25	29

## Supplementary Materials

The following are available online at https://www.mdpi.com/2076-2607/8/5/653/s1, Table S1: List of oomycete genomes used in this study, Table S2: Repetitive element annotation, Table S3: Functional annotation of gene models, Table S4: Annotation of mitochondrial genes, Table S5: Annotation of RxLR effectors, Table S6: Annotation of CAZymes using dbCAN2, Table S7: List of clusters of tandemly duplicated genes, Table S8: List of identified extracellular protein groups, Table S9: List of identified mycelial protein groups, Figure S1: Nucleotide alignment of *Phytophthora* mitochondrial genomes, Figure S2: Distribution of CAZyme families across 44 oomycete genomes.
