# Supplementary material for: Comparative Genomic and Proteomic Analyses of Three Widespread Phytophthora Species: Phytophthora chlamydospora, Phytophthora gonapodyides and Phytophthora pseudosyringae
Source: Microorganisms. 2020 Apr 30;8(5):653. doi: 10.3390/microorganisms8050653 (PMC7285336; doi:10.3390/microorganisms8050653)
Supplement: Supplementary file 1 [file microorganisms-08-00653-s001.zip › microorganisms-782621-supplementary/Supplementary Table S1. Oomycete genomes used in this study.docx]

**Supplementary Table 1. Genomic data used in this study**

| **Species** | **CAZyme Analysis** | **Gene Prediction Database** | **Phylogenomics** | **Reference** |
| --- | --- | --- | --- | --- |
| *Albugo candida* | X |  |  | (Links et al., 2011) |
| *Albugo laibachii* | X |  |  | (Kemen et al., 2011) |
| *Aphanomyces astaci* | X |  |  | PRJNA264335 |
| *Aphanomyces invadans* | X |  |  | PRJNA188082 |
| *Bremia lactucae* | X | X | X | (Fletcher et al., 2019) |
| *Hyaloperonospora arabidopsidis* | X | X | X | (Baxter et al., 2010) |
| *Peronospora belbahrii* |  |  | X | (Thines et al., 2019) |
| *Peronospora effusa* | X | X | X | (Fletcher et al., 2018) |
| *Phytophthora agathidicida* | X |  | X | (Studholme et al., 2015) |
| *Phytophthora cactorum* | X | X | X | (Armitage et al., 2018) |
| *Phytophthora capsici* | X | X | X | (Lamour et al., 2012) |
| *Phytophthora chlamydospora* | X |  | X | This study |
| *Phytophthora cinnamomi* | X |  | X | (Studholme et al., 2015) |
| *Phytophthora colocasiae* |  |  | X | (Vetukuri et al., 2018a) |
| *Phytophthora cryptogea* | X |  |  | (Feau et al., 2016) |
| *Phytophthora fragariae* | X |  | X | (Adams et al., 2019) |
| *Phytophthora gonapodyides* | X |  | X | This study |
| *Phytophthora infestans* | X | X | X | (Haas et al., 2009) |
| *Phytophthora kernoviae* | X |  | X | (Studholme et al., 2019) |
| *Phytophthora lateralis* |  |  | X | (Feau et al., 2016) |
| *Phytophthora litchii* |  |  | X | (Ye et al., 2016) |
| *Phytophthora megakarya* | X | X | X | (Ali et al., 2017) |
| *Phytophthora multivora* | X |  | X | (Studholme et al., 2015) |
| *Phytophthora nicotianae* | X |  | X | (Liu et al., 2016) |
| *Phytophthora palmivora* | X | X |  | (Ali et al., 2017) |
| *Phytophthora parasitica* | X | X | X | PRJNA259235 |
| *Phytophthora pinifolia* | X |  | X | (Feau et al., 2016) |
| *Phytophthora pisi* | X |  | X | PRJEB6298 |
| *Phytophthora plurivora* | X | X | X | (Vetukuri et al., 2018b) |
| *Phytophthora pluvialis* | X |  | X | (Studholme et al., 2015) |
| *Phytophthora pseudosyringae* | X |  | X | This study |
| *Phytophthora ramorum* | X | X | X | (Tyler et al., 2006) |
| *Phytophthora rubi* | X |  | X | (Tabima et al., 2017) |
| *Phytophthora sojae* | X | X | X | (Tyler et al., 2006) |
| *Phytophthora taxon totara* | X |  | X | (Studholme et al., 2015) |
| *Phytopythium vexans* | X |  |  | (Adhikari et al., 2013) |
| *Pilasporangium apinafurcum* | X |  |  | (Uzuhashi et al., 2017) |
| *Plasmopara halstedii* | X | X | X | (Sharma et al., 2015) |
| *Plasmopara muralis* | X |  | X | (Dussert et al., 2019) |
| *Plasmopara viticola* |  | X | X | (Dussert et al., 2019) |
| *Pseudoperonospora cubensis* |  |  | X | PRJNA80635 |
| *Pythium aphanidermatum* | X |  |  | (Adhikari et al., 2013) |
| *Pythium arrhenomanes* | X |  |  | (Adhikari et al., 2013) |
| *Pythium insidiosum* | X |  |  | (Rujirawat et al., 2015) |
| *Pythium irregulare* | X |  |  | (Adhikari et al., 2013) |
| *Pythium iwayamai* | X |  |  | (Adhikari et al., 2013) |
| *Pythium oligandrum* | X |  |  | (Kushwaha et al., 2017) |
| *Pythium ultimum* | X |  | X | (Lévesque et al., 2010) |
| *Saprolegnia diclina* | X |  |  | PRJNA86859 |
| *Saprolegnia parasitica* | X |  |  | (Jiang et al., 2013) |

**References**

Adams, T.M., Armitage, A.D., Sobczyk, M.K., Bates, H.J., Tabima, J.F., Kronmiller, B.A., Tyler, B.M., Grünwald, N.J., Dunwell, J.M., Nellist, C.F., Harrison, R.J., 2019. Genomic investigation of the strawberry pathogen &lt;em&gt;Phytophthora fragariae&lt;/em&gt; indicates pathogenicity is determined by transcriptional variation in three key races. bioRxiv 860619.

Adhikari, B.N., Hamilton, J.P., Zerillo, M.M., Tisserat, N., Lévesque, C.A., Buell, C.R., 2013. Comparative Genomics Reveals Insight into Virulence Strategies of Plant Pathogenic Oomycetes. PLoS One 8.

Ali, S.S., Shao, J., Lary, D.J., Kronmiller, B.A., Shen, D., Strem, M.D., Amoako-Attah, I., Akrofi, A.Y., Begoude, B.A.D., ten Hoopen, G.M., Coulibaly, K., Kebe, B.I., Melnick, R.L., Guiltinan, M.J., Tyler, B.M., Meinhardt, L.W., Bailey, B.A., 2017. Phytophthora megakarya and Phytophthora palmivora, Closely Related Causal Agents of Cacao Black Pod Rot, Underwent Increases in Genome Sizes and Gene Numbers by Different Mechanisms. Genome Biol. Evol. 9, 536–557.

Armitage, A.D., Lysøe, E., Nellist, C.F., Lewis, L.A., Cano, L.M., Harrison, R.J., Brurberg, M.B., 2018. Bioinformatic characterisation of the effector repertoire of the strawberry pathogen Phytophthora cactorum. PLoS One 13, 1–24.

Baxter, L., Tripathy, S., Ishaque, N., Boot, N., Cabral, A., Kemen, E., Thines, M., Ah-Fong, A., Anderson, R., Badejoko, W., Bittner-Eddy, P., Boore, J.L., Chibucos, M.C., Coates, M., Dehal, P., Delehaunty, K., Dong, S., Downton, P., Dumas, B., Fabro, G., Fronick, C., Fuerstenberg, S.I., Fulton, L., Gaulin, E., Govers, F., Hughes, L., Humphray, S., Jiang, R.H.Y., Judelson, H., Kamoun, S., Kyung, K., Meijer, H., Minx, P., Morris, P., Nelson, J., Phuntumart, V., Qutob, D., Rehmany, A., Rougon-Cardoso, A., Ryden, P., Torto-Alalibo, T., Studholme, D.J., Wang, Y., Win, J., Wood, J., Clifton, S.W., Rogers, J., Van den Ackerveken, G., Jones, J.D.G., McDowell, J.M., Beynon, J., Tyler, B.M., 2010. Signatures of Adaptation to Obligate Biotrophy in the Hyaloperonospora arabidopsidis Genome. Science (80-. ). 330, 1549–1551.

Dussert, Y., Mazet, I.D., Couture, C., Gouzy, J., Piron, M.-C., Kuchly, C., Bouchez, O., Rispe, C., Mestre, P., Delmotte, F., 2019. A High-Quality Grapevine Downy Mildew Genome Assembly Reveals Rapidly Evolving and Lineage-Specific Putative Host Adaptation Genes. Genome Biol. Evol. 11, 954–969.

Feau, N., Taylor, G., Dale, A.L., Dhillon, B., Bilodeau, G.J., Birol, I., Jones, S.J.M., Hamelin, R.C., 2016. Genome sequences of six Phytophthora species threatening forest ecosystems. Genomics Data 10, 85–88.

Fletcher, K., Gil, J., Bertier, L.D., Kenefick, A., Wood, K.J., Zhang, L., Reyes-Chin-Wo, S., Cavanaugh, K., Tsuchida, C., Wong, J., Michelmore, R., 2019. Genomic signatures of heterokaryosis in the oomycete pathogen Bremia lactucae. Nat. Commun. 10, 1–13.

Fletcher, K., Klosterman, S.J., Derevnina, L., Martin, F., Bertier, L.D., Koike, S., Reyes-Chin-Wo, S., Mou, B., Michelmore, R., 2018. Comparative genomics of downy mildews reveals potential adaptations to biotrophy. BMC Genomics 19, 8–10.

Haas, B.J., Kamoun, S., Zody, M.C., Jiang, R.H.Y., Handsaker, R.E., Cano, L.M., Grabherr, M., Kodira, C.D., Raffaele, S., Torto-Alalibo, T., Bozkurt, T.O., Ah-Fong, A.M. V., Alvarado, L., Anderson, V.L., Armstrong, M.R., Avrova, A., Baxter, L., Beynon, J., Boevink, P.C., Bollmann, S.R., Bos, J.I.B., Bulone, V., Cai, G., Cakir, C., Carrington, J.C., Chawner, M., Conti, L., Costanzo, S., Ewan, R., Fahlgren, N., Fischbach, M.A., Fugelstad, J., Gilroy, E.M., Gnerre, S., Green, P.J., Grenville-Briggs, L.J., Griffith, J., Grünwald, N.J., Horn, K., Horner, N.R., Hu, C.-H., Huitema, E., Jeong, D.-H., Jones, A.M.E., Jones, J.D.G., Jones, R.W., Karlsson, E.K., Kunjeti, S.G., Lamour, K., Liu, Z., Ma, L., MacLean, D., Chibucos, M.C., McDonald, H., McWalters, J., Meijer, H.J.G., Morgan, W., Morris, P.F., Munro, C.A., O’Neill, K., Ospina-Giraldo, M., Pinzón, A., Pritchard, L., Ramsahoye, B., Ren, Q., Restrepo, S., Roy, S., Sadanandom, A., Savidor, A., Schornack, S., Schwartz, D.C., Schumann, U.D., Schwessinger, B., Seyer, L., Sharpe, T., Silvar, C., Song, J., Studholme, D.J., Sykes, S., Thines, M., van de Vondervoort, P.J.I., Phuntumart, V., Wawra, S., Weide, R., Win, J., Young, C., Zhou, S., Fry, W., Meyers, B.C., van West, P., Ristaino, J., Govers, F., Birch, P.R.J., Whisson, S.C., Judelson, H.S., Nusbaum, C., 2009. Genome sequence and analysis of the Irish potato famine pathogen Phytophthora infestans. Nature 461, 393–398.

Jiang, R.H.Y., de Bruijn, I., Haas, B.J., Belmonte, R., Löbach, L., Christie, J., van den Ackerveken, G., Bottin, A., Bulone, V., Díaz-Moreno, S.M., Dumas, B., Fan, L., Gaulin, E., Govers, F., Grenville-Briggs, L.J., Horner, N.R., Levin, J.Z., Mammella, M., Meijer, H.J.G., Morris, P., Nusbaum, C., Oome, S., Phillips, A.J., van Rooyen, D., Rzeszutek, E., Saraiva, M., Secombes, C.J., Seidl, M.F., Snel, B., Stassen, J.H.M., Sykes, S., Tripathy, S., van den Berg, H., Vega-Arreguin, J.C., Wawra, S., Young, S.K., Zeng, Q., Dieguez-Uribeondo, J., Russ, C., Tyler, B.M., van West, P., 2013. Distinctive Expansion of Potential Virulence Genes in the Genome of the Oomycete Fish Pathogen Saprolegnia parasitica. PLoS Genet. 9, e1003272.

Kemen, E., Gardiner, A., Schultz-Larsen, T., Kemen, A.C., Balmuth, A.L., Robert-Seilaniantz, A., Bailey, K., Holub, E., Studholme, D.J., MacLean, D., Jones, J.D.G., 2011. Gene gain and loss during evolution of obligate parasitism in the white rust pathogen of Arabidopsis thaliana. PLoS Biol. 9.

Kushwaha, S.K., Vetukuri, R.R., Grenville-Briggs, L.J., 2017. Draft Genome Sequence of the Mycoparasitic Oomycete Pythium oligandrum Strain CBS 530.74. Genome Announc. 5, e00556-18.

Lamour, K.H., Mudge, J., Gobena, D., Hurtado-Gonzales, O.P., Schmutz, J., Kuo, A., Miller, N.A., Rice, B.J., Raffaele, S., Cano, L.M., Bharti, A.K., Donahoo, R.S., Finley, S., Huitema, E., Hulvey, J., Platt, D., Salamov, A., Savidor, A., Sharma, R., Stam, R., Storey, D., Thines, M., Win, J., Haas, B.J., Dinwiddie, D.L., Jenkins, J., Knight, J.R., Affourtit, J.P., Han, C.S., Chertkov, O., Lindquist, E.A., Detter, C., Grigoriev, I. V, Kamoun, S., Kingsmore, S.F., 2012. Genome sequencing and mapping reveal loss of heterozygosity as a mechanism for rapid adaptation in the vegetable pathogen Phytophthora capsici. Mol. Plant-Microbe Interact. 25, 1350–1360.

Lévesque, C.A., Brouwer, H., Cano, L., Hamilton, J.P., Holt, C., Huitema, E., Raffaele, S., Robideau, G.P., Thines, M., Win, J., Zerillo, M.M., Beakes, G.W., Boore, J.L., Busam, D., Dumas, B., Ferriera, S., Fuerstenberg, S.I., Gachon, C.M., Gaulin, E., Govers, F., Grenville-Briggs, L., Horner, N., Hostetler, J., Jiang, R.H., Johnson, J., Krajaejun, T., Lin, H., Meijer, H.J., Moore, B., Morris, P., Phuntmart, V., Puiu, D., Shetty, J., Stajich, J.E., Tripathy, S., Wawra, S., van West, P., Whitty, B.R., Coutinho, P.M., Henrissat, B., Martin, F., Thomas, P.D., Tyler, B.M., De Vries, R.P., Kamoun, S., Yandell, M., Tisserat, N., Buell, C.R., 2010. Genome sequence of the necrotrophic plant pathogen Pythium ultimum reveals original pathogenicity mechanisms and effector repertoire. Genome Biol. 11, R73.

Links, M.G., Holub, E., Jiang, R.H., Sharpe, A.G., Hegedus, D., Beynon, E., Sillito, D., Clarke, W.E., Uzuhashi, S., Borhan, M.H., 2011. De novo sequence assembly of Albugo candida reveals a small genome relative to other biotrophic oomycetes. BMC Genomics 12, 503.

Liu, H., Ma, X., Yu, H., Fang, D., Li, Y., Wang, X., Wang, W., Dong, Y., Xiao, B., 2016. Genomes and virulence difference between two physiological races of Phytophthora nicotianae. Gigascience 5, 3.

Rujirawat, T., Patumcharoenpol, P., Lohnoo, T., Yingyong, W., Lerksuthirat, T., Tangphatsornruang, S., Suriyaphol, P., Grenville-Briggs, L.J., Garg, G., Kittichotirat, W., Krajaejun, T., 2015. Draft Genome Sequence of the Pathogenic Oomycete Pythium insidiosum Strain Pi-S, Isolated from a Patient with Pythiosis. Genome Announc. 3, e00574-15.

Sharma, R., Xia, X., Cano, L.M., Evangelisti, E., Kemen, E., Judelson, H., Oome, S., Sambles, C., van den Hoogen, D.J., Kitner, M., Klein, J., Meijer, H.J.G., Spring, O., Win, J., Zipper, R., Bode, H.B., Govers, F., Kamoun, S., Schornack, S., Studholme, D.J., Van den Ackerveken, G., Thines, M., 2015. Genome analyses of the sunflower pathogen Plasmopara halstedii provide insights into effector evolution in downy mildews and Phytophthora. BMC Genomics 16, 741.

Studholme, D.J., McDougal, R.L., Sambles, C., Hansen, E., Hardy, G., Grant, M., Ganley, R.J., Williams, N.M., 2015. Genome sequences of six Phytophthora species associated with forests in New Zealand. Genomics Data 7, 54–56.

Studholme, D.J., Panda, P., Sanfuentes Von Stowasser, E., González, M., Hill, R., Sambles, C., Grant, M., Williams, N.M., McDougal, R.L., 2019. Genome sequencing of oomycete isolates from Chile supports the New Zealand origin of Phytophthora kernoviae and makes available the first Nothophytophthora sp. genome. Mol. Plant Pathol. 20, 423–431.

Tabima, J.F., Kronmiller, B.A., Press, C.M., Tyler, B.M., Zasada, I.A., Grünwald, N.J., 2017. Whole Genome Sequences of the Raspberry and Strawberry Pathogens Phytophthora rubi and P. fragariae. Mol. Plant-Microbe Interact. 30, 767–769.

Thines, M., Sharma, R., Rodenburg, S.Y.A., Gogleva, A., Judelson, H.S., Xia, X., Hoogen, J. van den, Kitner, M., Klein, J., Neilen, M., Ridder, D. de, Seidl, M.F., Ackerveken, G. Van den, Govers, F., Schornack, S., Studholme, D.J., 2019. The genome of Peronospora belbahrii reveals high heterozygosity, a low number of canonical effectors and CT-rich promoters. bioRxiv 721027.

Tyler, B.M., Tripathy, S., Zhang, X., Dehal, P., Jiang, R.H.Y., Aerts, A., Arredondo, F.D., Baxter, L., Bensasson, D., Beynon, J.L., Chapman, J., Damasceno, C.M.B., Dorrance, A.E., Dou, D., Dickerman, A.W., Dubchak, I.L., Garbelotto, M., Gijzen, M., Gordon, S.G., Govers, F., Grunwald, N.J., Huang, W., Ivors, K.L., Jones, R.W., Kamoun, S., Krampis, K., Lamour, K.H., Lee, M.-K., McDonald, W.H., Medina, M., Meijer, H.J.G., Nordberg, E.K., Maclean, D.J., Ospina-Giraldo, M.D., Morris, P.F., Phuntumart, V., Putnam, N.H., Rash, S., Rose, J.K.C., Sakihama, Y., Salamov, A.A., Savidor, A., Scheuring, C.F., Smith, B.M., Sobral, B.W.S., Terry, A., Torto-Alalibo, T.A., Win, J., Xu, Z., Zhang, H., Grigoriev, I. V, Rokhsar, D.S., Boore, J.L., 2006. Phytophthora genome sequences uncover evolutionary origins and mechanisms of pathogenesis. Science 313, 1261–6.

Uzuhashi, S., Endoh, R., Manabe, R., Ohkuma, M., 2017. Draft Genome Sequences of the Oomycete Pilasporangium apinafurcum Strains JCM 30513 and JCM 30514, Formerly Classified as Pythium apinafurcum. Genome Announc. 5, 4–5.

Vetukuri, R.R., Kushwaha, S.K., Sen, D., Whisson, S.C., Lamour, K.H., Grenville-Briggs, L.J., 2018a. Genome sequence resource for the oomycete taro pathogen phytophthora colocasiae. Mol. Plant-Microbe Interact. 31, 903–905.

Vetukuri, R.R., Tripathy, S., Mathu, M.C., Panda, A., Kushwaha, S.K., Chawade, A., Andreasson, E., Grenville-Briggs, L.J., Whisson, S.C., 2018b. Draft genome sequence for the tree pathogen phytophthora plurivora. Genome Biol. Evol. 10, 2432–2442.

Ye, W., Wang, Yang, Shen, D., Li, D., Pu, T., Jiang, Z., Zhang, Z., Zheng, X., Tyler, B.M., Wang, Yuanchao, 2016. Sequencing of the Litchi Downy Blight Pathogen Reveals It Is a Phytophthora Species With Downy Mildew-Like Characteristics. Mol. Plant-Microbe Interact. 29, 573–583.
