# Supplementary figures and images for: Comparative Genomic and Proteomic Analyses of Three Widespread Phytophthora Species: Phytophthora chlamydospora, Phytophthora gonapodyides and Phytophthora pseudosyringae
Source: Microorganisms. 2020 Apr 30;8(5):653. doi: 10.3390/microorganisms8050653 (PMC7285336; doi:10.3390/microorganisms8050653)

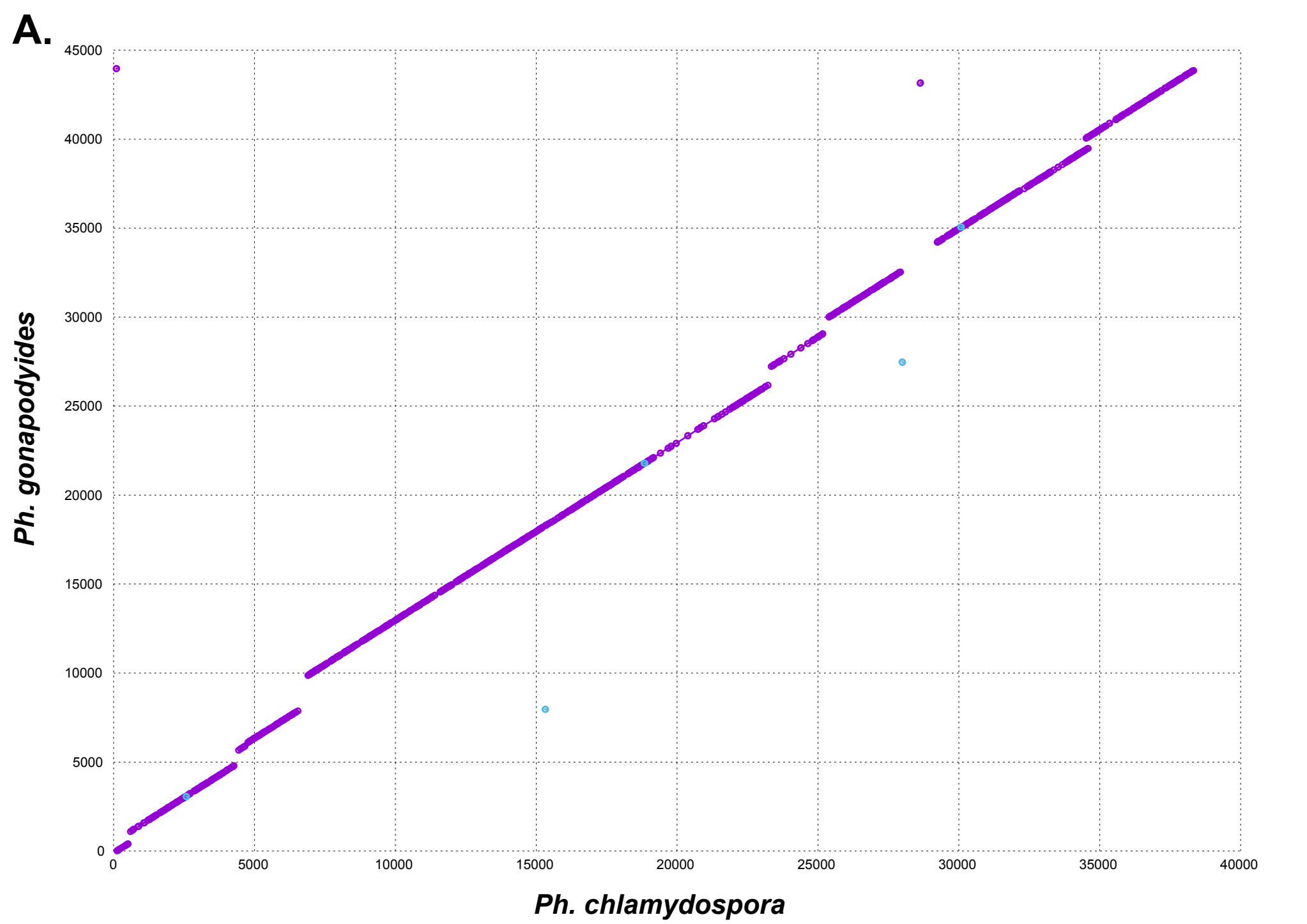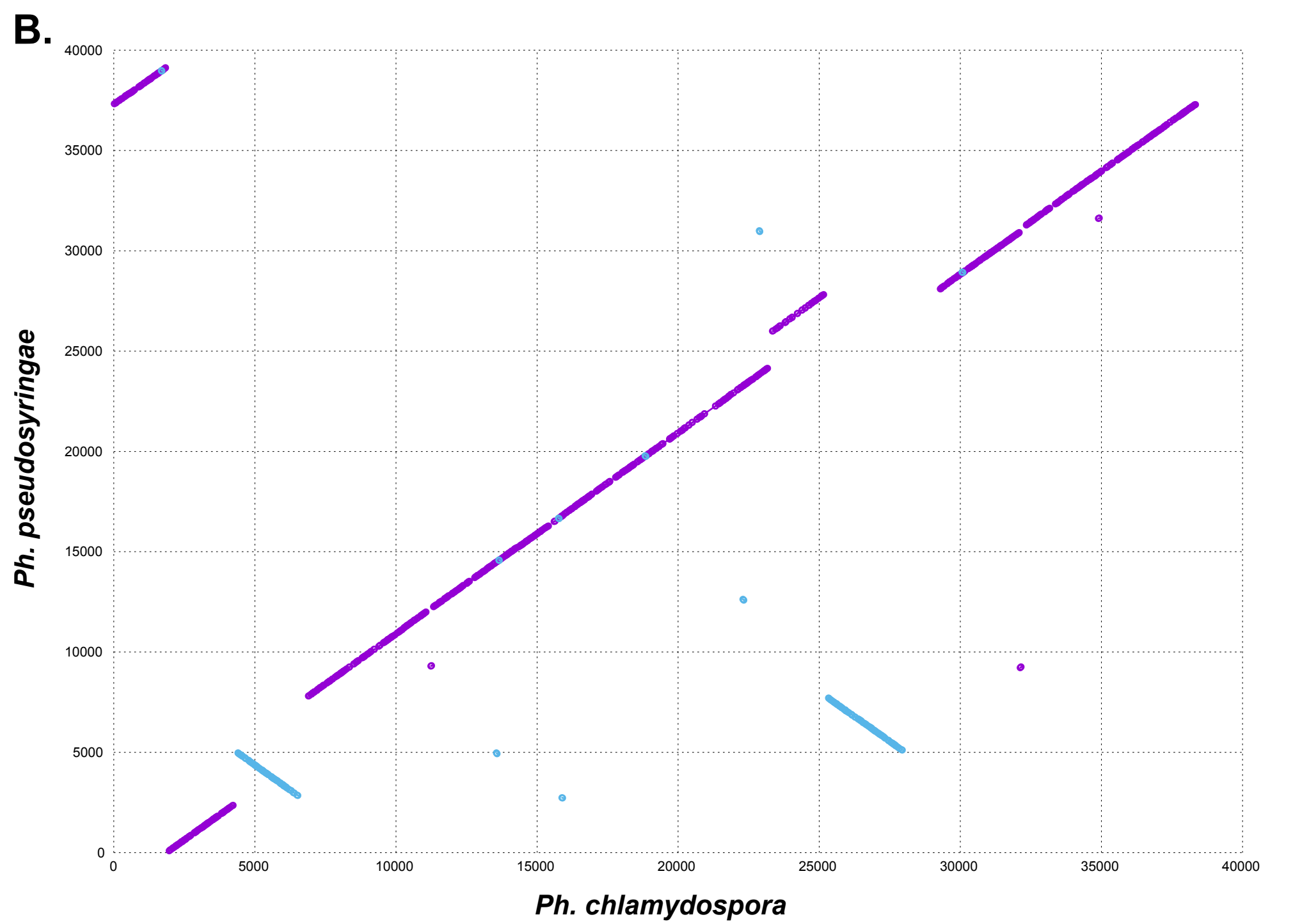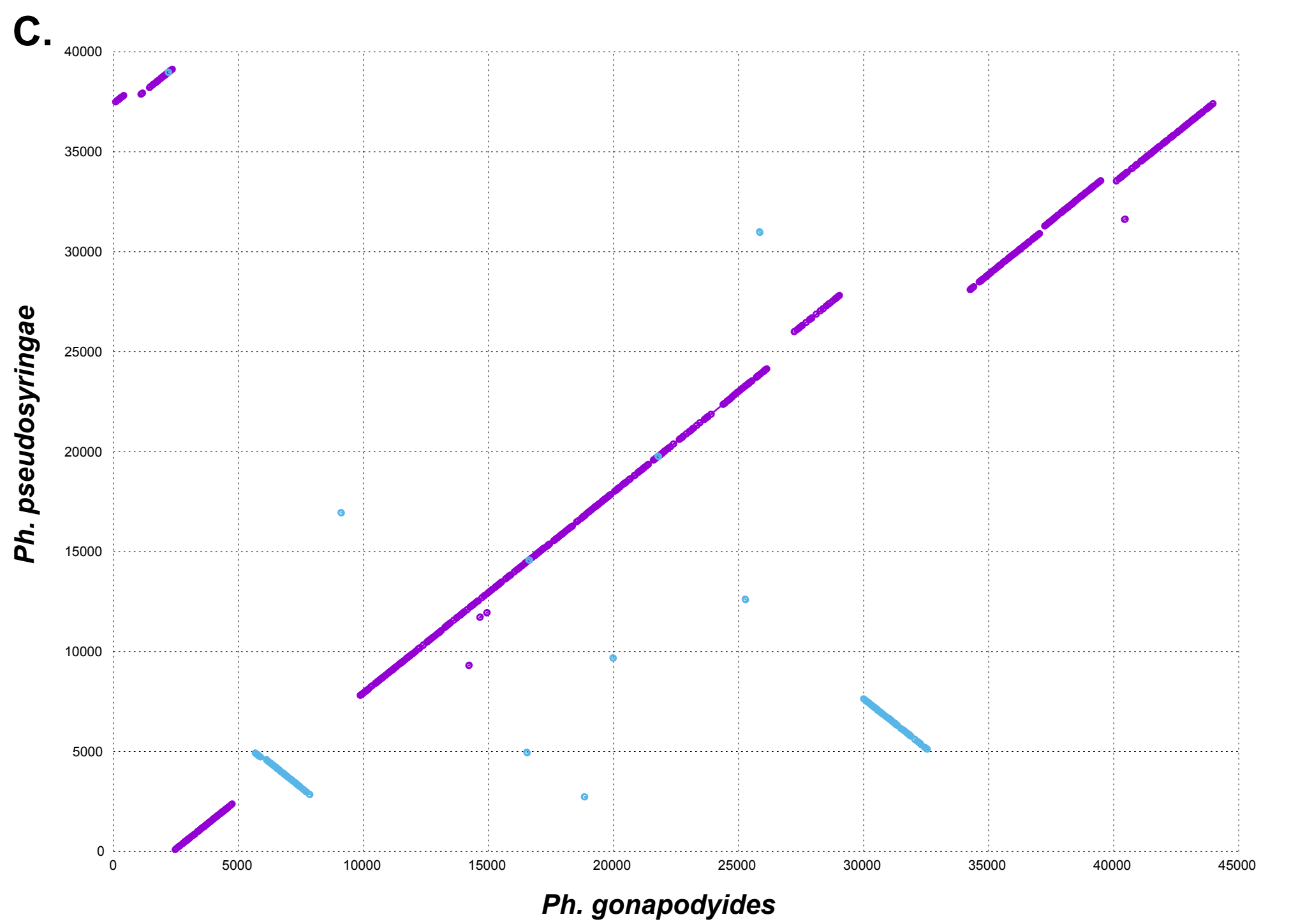

Supplement: Supplementary file 1 [file microorganisms-08-00653-s001.zip › microorganisms-782621-supplementary/Supplementary Figure S1. Nucleotide Alignments of Phytophthora Mitochondrial Genomes.pdf]
